# Effectiveness of community health workers delivering preventive interventions for maternal and child health in low- and middle-income countries: a systematic review

**DOI:** 10.1186/1471-2458-13-847

**Published:** 2013-09-13

**Authors:** Brynne Gilmore, Eilish McAuliffe

**Affiliations:** 1 Centre for Global Health, Trinity College, Dublin, Ireland

**Keywords:** Community health workers, Maternal and child health, Low-and middle-income countries, Prevention, Intervention, Human resources for health

## Abstract

**Background:**

Community Health Workers are widely utilised in low- and middle-income countries and may be an important tool in reducing maternal and child mortality; however, evidence is lacking on their effectiveness for specific types of programmes, specifically programmes of a preventive nature. This review reports findings on a systematic review analysing effectiveness of preventive interventions delivered by Community Health Workers for Maternal and Child Health in low- and middle-income countries.

**Methods:**

A search strategy was developed according to the Evidence for Policy and Practice Information and Co-ordinating Centre’s (EPPI-Centre) guidelines and systematic searching of the following databases occurred between June 8 – 11^th^, 2012: CINAHL, Embase, Ovid Nursing Database, PubMed, Scopus, Web of Science and POPLINE. Google, Google Scholar and WHO search engines, as well as relevant systematic reviews and reference lists from included articles were also searched. Inclusion criteria were: i) Target beneficiaries should be pregnant or recently pregnant women and/or children under-5 and/or caregivers of children under-5; ii) Interventions were required to be preventive and delivered by Community Health Workers at the household level. No exclusion criteria were stipulated for comparisons/controls or outcomes. Study characteristics of included articles were extracted using a data sheet and a peer tested quality assessment. A narrative synthesis of included studies was compiled with articles being coded descriptively to synthesise results and draw conclusions.

**Results:**

A total of 10,281 studies were initially identified and through the screening process a total of 17 articles detailing 19 studies were included in the review. Studies came from ten different countries and consisted of randomized controlled trials, cluster randomized controlled trials, before and after, case control and cross sectional studies. Overall quality of evidence was found to be moderate. Five main preventive intervention categories emerged: malaria prevention, health education, breastfeeding promotion, essential newborn care and psychosocial support. All categories showed some evidence for the effectiveness of Community Health Workers; however they were found to be especially effective in promoting mother-performed strategies (skin to skin care and exclusive breastfeeding).

**Conclusions:**

Community Health Workers were shown to provide a range of preventive interventions for Maternal and Child Health in low- and middle-income countries with some evidence of effective strategies, though insufficient evidence is available to draw conclusions for most interventions and further research is needed.

## Background

There has been insufficient progress in many low- and middle-income countries (LMIC) towards reducing maternal mortality by 75% and under-5 deaths by two thirds – the targets for Millennium Development Goals 5 and 4, respectively. In 2010 alone 287,000 women and 7.6 million children under-5 died due to pregnancy related complications and a lack of adequate health care [1,2]. Though evidence-based cost-effective interventions that are predicted to prevent up to one third of Maternal and Child Health (MCH) complications and deaths with universal coverage have been identified [3-6], over 50% of children in under-resourced areas do not have access to these simple interventions [7].

Ninety nine per cent of maternal and child deaths occur in low- and middle-income countries where there is a severe shortage of human resources for health (HRH), which is one of the most significant constraints to achieving Millennium Development Goals (MDGs) 4 and 5 [8-10]. Identified as a distinguishing feature of providing primary care for individuals in resource poor settings in the Alma-Ata Declaration 1978 [11], Community Health Workers (CHWs) act as a mitigating factor to the HRH crisis by providing essential MCH care at the household and community level, reducing inequalities in health care for marginalized populations, providing education and mainly curative health services, and having the essential role of liaising between the community and more skilled workers and facility-based services [12-14].

Systematic reviews examining CHW programmes worldwide found that these cadres are effective in reducing maternal, neonatal and child mortality in resource poor settings; however, evidence on the effectiveness of different programme types was recognised as lacking, particularly evidence from prevention programmes [14,15]. An analysis of the success of CHW programmes delivering curative interventions for children in sub-Saharan Africa documented large mortality reductions with malaria [10], and indicated the need for further investigation into programme effectiveness in LMICs.

This article reports on findings from a systematic review of studies evaluating the effectiveness of exclusively preventive interventions for MCH delivered by CHWs in LMIC at the household level (Table 1). Prior to the review, an advisory committee was formed as a means of ensuring quality and reducing bias throughout the stages of the review [16-18]. A global health professional, a Cochrane-trained researcher with expertise in identifying and disseminating information on evidence-based interventions and a Health Sciences Search Librarian were consulted for assistance in topic formulation and article screening, format and scope of review, and appropriate bibliographic databases and search terms, respectively.

**Table 1:** Definition of terms

**Term**	**Explanation**
**Maternal Health**	Refers to the health of a woman during pregnancy or within 42 days of termination of pregnancy, due to pregnancy related issues [2]
**Child Health**	Refers to any health issues in children ages five years or less
**Community Health Worker**	Defined as “…members of the communities where they work, should be selected by the communities, should be answerable to the communities for their activities, should be supported by the health system but not necessarily a part of its organization, and have shorter training than professional workers” [19]
**Prevention**	Interventions or “measures adopted by or practiced on persons not currently feeling the effects of a disease [or negative health outcome], intended to decrease the risk that that disease [or negative health outcomes] will afflict them in the future” [20]

## Methods

### Review format

This review has followed the Evidence for Policy and Practice Information and Co-ordinating Centre’s (EPPI-Centre) Methods for Conducting a Systematic Review [18]. The EPPI format allows for a greater variety of study designs to be included, as well as a mixed-methods synthesis when appropriate [17] whereas traditional systematic reviews (e.g. Cochrane method) include only Randomised Controlled Trials (RCTs) and are interested in combining numerical data in the form of a meta-analysis for data synthesis [21]. The EPPI-Centre focuses on social science and public policy reviews, and like other systematic review institutes, requires accountability, rigour and explicit methods in conducting a review. There is no review protocol of this review.

A narrative synthesis is used to present and analyse findings. As this review includes both experimental and observational studies with large heterogeneity in interventions and measures, a statistical analysis is inappropriate. When reviews include studies that cannot be combined statistically but are still undertaken with the same amount of rigour and quality, they are classified as qualitative systematic reviews [21].

### Search strategy

Due to the diversity of potential interventions, populations, study types and outcomes, a multi-stage search strategy was developed to identify relevant publications. The search terminology was adapted, with permission, from Lewin et al. [15], which included the methodological component of Cochrane’s Effective Practice and Organisation of Care Group (EPOC) guidelines, combined with free text and key terms. The Lewin et al. [15] search terminology was then adjusted to be more relevant to this study’s research question by adding both key terms and free text terms relating to the topic that were identified by various scoping searches, see Additional file 1. Alternative names for Community Health Workers, see Additional file 2, identified through various literature sources, were added.

As recommended by the EPPI-Centre [18] the strategy attempted to balance sensitivity with specificity in its results; however, due to the large amount of heterogeneity in inclusion criteria the strategy was quite sensitive (i.e. produced large quantities of articles irrelevant to the topic). The finalized search strategy was then modified to fit with the different electronic databases’ nomenclature. The following databases were searched from June 8 – 11^th^, 2012: CINAHL, Embase, Ovid Nursing Database, PubMed, Scopus, Web of Science and POPLINE. In addition, the Google and Google Scholar search engines, as well as The World Health Organization’s website were searched for relevant articles on June 11^th^, 2012. Reference lists from other related systematic reviews [10,11,14,15] were also searched as well as the references from articles that were identified for inclusion in the review [22-38]. Article screening was conducted in several stages by one reviewer with the assistance of a second reviewer in the final stage.

### Inclusion and exclusion criteria

*Target beneficiaries* include women pregnant to 42 days post termination, children under 5 and primary caregivers of children under 5 who have the ability to influence the child’s health.

*Interventions* were required to be preventive [20] and have been delivered at the household level by Community Health Workers, using the WHO Study Group [39] definition of “…members of the communities where they work, should be selected by the communities, should be answerable to the communities for their activities, should be supported by the health system but not necessarily a part of its organization, and have shorter training than professional workers”.

No restrictions on outcome or study design were included. Due to the varying types of anticipated study designs, no restrictions were imposed on the control or comparison group. Only studies conducted in low- and middle-income countries, as identified by The World Bank at time of study initiation, and only those articles published from 1990 to present were included. This time period was chosen to coincide with the re-emergence of the popularity of CHW programmes [11], to be consistent with the MDG timeframe and for scoping feasibility due do this study’s resource restriction. To limit bias in both intervention areas and research publication sites, there was no language restriction. Non-English papers’ abstracts were reviewed provided they were available in English and assessed for inclusion. The decision was taken to translate non-English papers, however this was unnecessary as none fit the inclusion criteria upon abstract review.

Articles were excluded if they did not meet the inclusion criteria or: if interventions were not clear or in studies with multiple intervention techniques where it was not possible to separate out specific preventive intervention outcomes; if the description of CHWs was insufficient or their role in the intervention was ill-defined; and if multiple health cadres were responsible for the intervention’s implementation and the CHW’s specific role could not be discerned, see Additional file 3.

### Study quality assessment

Due to the scope of study designs included in this review, which may affect quality rating, and the lack of a meta-analysis, no studies were excluded based on the quality assessment. Studies were empirically rated using Effective Public Health Practice Project (EPHPP)’s Quality Assessment Tool for Quantitative Studies [40] which uses a generic scale to evaluate a range of study designs and has been independently evaluated and judged as suitable for use in article appraisal for systematic reviews [19,41].

### Data extraction and synthesis

Data was extracted systematically using a pre-formulated tool consisting of: setting, study design, population, intervention, control/comparison, group allocation methods, outcomes and quality rating, see Additional file 4. Data was then synthesised qualitatively by combining studies with similarities in interventions. Articles were subsequently coded descriptively by the reviewer to synthesise the results and draw conclusions.

## Results

**Figure 1:**
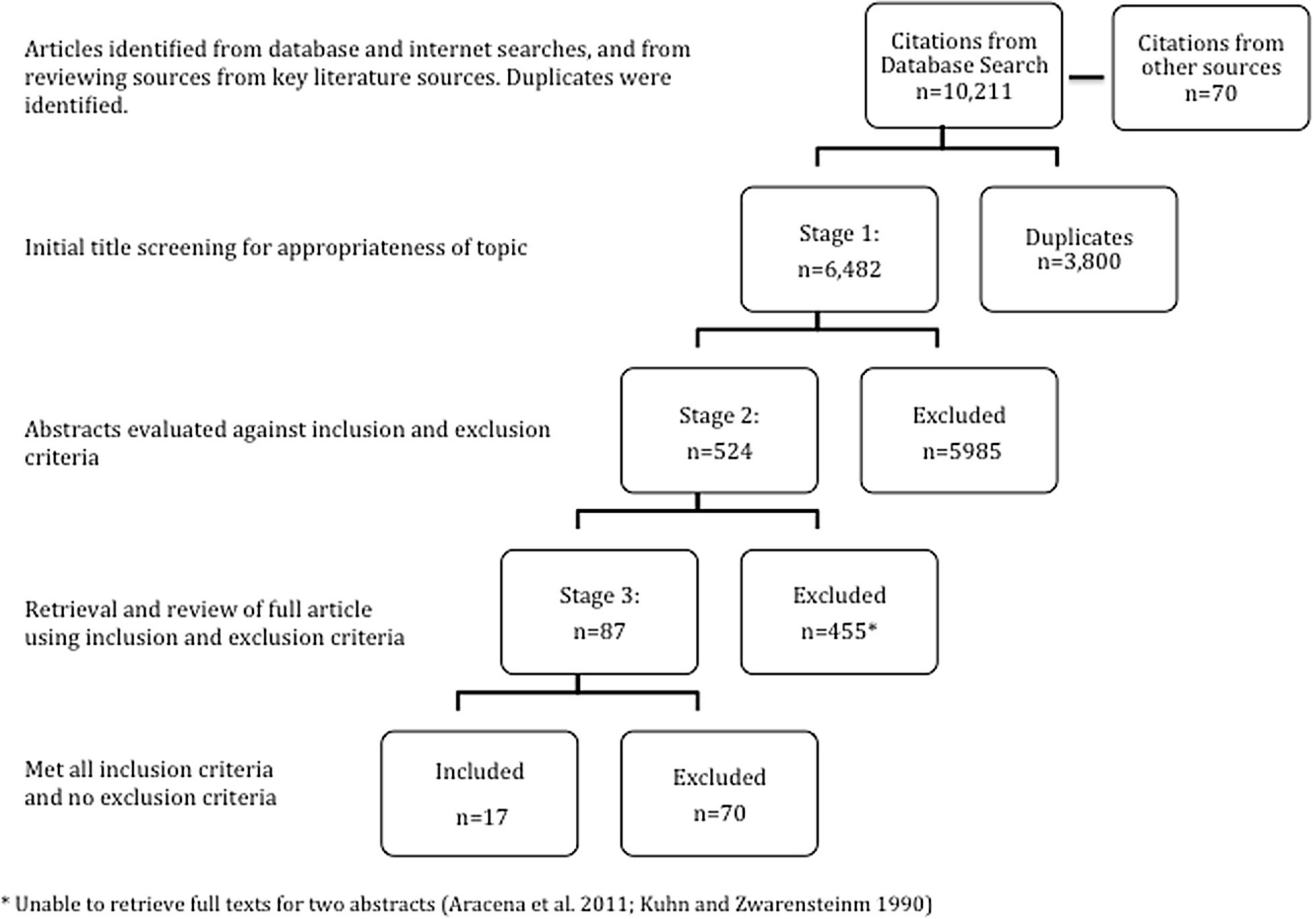
Article screening.

A total of 10,281 titles from 1990 to present were identified from the database search and other sources, of which 3,800 were duplicates. See Additional file 5 for the search log. Full texts of 87 studies were assessed (the full texts of 2 studies could not be retrieved) and 70 were excluded for a variety of reasons, see Additional file 6 for characteristics of excluded studies. Upon screening process completion, Figure 1, seventeen articles comprising of 19 studies met the inclusion criteria. One paper, [22] examines an intervention across multiple countries (3) and reports results separately for each site. Therefore, a total of 19 primary studies are included in this review.

### Study characteristics

#### Setting

The majority of the included studies were conducted in South Asia, five in Bangladesh [23-27], three in India [28-30] and two in Pakistan [31,32]. Seven studies took place in Africa, two in Uganda [22,33] two in South Africa [22,34] and one each in Burkina Faso [22], Nigeria [35] and Ghana [36]. Of the remaining two studies, one was conducted in Mexico [37] and one in the Philippines [38].

#### Study design

Both experimental and descriptive studies were eligible to be included in the review. Of the studies included, eight are cluster-randomized controlled trials [22,25,27,30,32,37], four are randomized-controlled trials [23,31,34,38], five are before and after [28,29,33,35,36], one is cross-sectional [24] and one is a case series [26].

#### Year of publication

**Figure 2:**
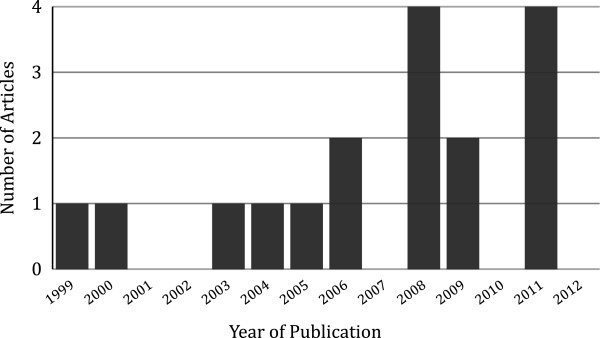
Article publication date.

Though the search identified articles published from 1990 to present, all included articles were published between 1999 and 2011. This trend is consistent with the decrease in CHW programmes throughout the 1990s and a more recent recognition of their potential contributions to health care in low resource settings [7,12]. Figure 2 presents the distribution of articles by year of publication.

#### CHW characteristics

There were large variations in community health worker prerequisites, recruitment, training, supervision and workload between the studies identified, with these characteristics documented in Additional file 7. Studies mainly varied in their use of pre-trained workers, education requirements, monetary compensation (if any), training schedules, occurrence of refresher training sessions, amount and quality of supervision, and workload represented by the ratio of CHWs per target beneficiary.

### Study quality assessment

The quality of each included study was assessed using Effective Public Health Practice Project’s (EPHPP) Quality Assessment Tool for Quantitative Studies. Studies were evaluated across eight categories (selection bias, study design, confounders, blinding, data collection methods, withdrawals and drops, intervention integrity, analyses) and given a score of 1 to 3 accordingly. Averaging the category rankings assigned an overall rating of weak, moderate or strong for 1, 2 or 3 respectively. Studies whose average was between two ratings were given a double-label to represent their standing. Seven studies were categorized as strong [22,23,30-32], four as moderate-strong [34-36,38], five as moderate [24,25,29,33,37] one as weak-moderate [27] and two as weak [26,28]. Articles were not excluded due to a low quality rating but this was considered with analysing effectiveness.

### Narrative synthesis

Heterogeneity in intervention designs and outcomes made quantitative methods, including meta-analysis and effect size for synthesis impossible and inappropriate. Studies were therefore categorised and described as reported in the following narrative synethesis.

#### Malarial interventions

Ahorulu and colleagues [36] conducted a before and after study in the perennial malaria endemic Volta region of Ghana. Community Health Workers were responsible for delivering Intermittent Preventative Treatment for Children (IPTc) 6 to 60 months every four months, for one year. In the 357 children evaluated one-year post intervention inception, malarial parasite prevalence was reduced from 25.0% to 3%. Rates of anaemia were also significantly reduced from 26.6% at baseline to 16.8% at final evaluation, and the use of insecticide treated bed-nets (ITNs) increased from 38.5% to 60%.

In Akwa Ibom, Nigeria, Okeibunor et al. [35] utilized a before and after parallel group design for analyzing the extent to which community based interventions can improve malaria prevention during pregnancy. Community Health Workers distributed two doses of sulphadoxine-pyrimethamine (SP) for Intermittent Preventative Treatment in Pregnancy (IPTp) and insecticide treated bed-nets, as well as providing basic counseling services. Compared to the endline control group (n=627), an additional 7.4% of women in the intervention group (n=751) utilized a bed-net during pregnancy, and an additional 8.5% slept under an ITN postnatally. The intervention group also had significantly higher rates of taking at least two doses of SP, with the fraction of women increasing by 35.5% compared to the control group. No difference in ANC visits was observed between intervention and control groups.

#### Health education interventions

Three studies covering interventions on food safety, immunization promotion and overall child health and safety were found. A before and after study by Sheth and Obran [28] in an urban Indian slum promoted food safety education to mothers of underprivileged children between 6 and 24 months. This intervention involved CHWs conducting home visits using visual educational materials, to 200 low-income households purposely selected across 8 randomly chosen Anganwai centre catchment areas. Pre and post intervention analysis found a 52.5% reduction in child diarrhoea, 65% and 10% reduction in mother’s and children’s microbial load, respectively, indicating improved hand-washing behaviour. Inappropriate environmental sanitation and mother’s poor hygiene practices decreased by 36.5% and 8.5%, respectively. As well, there was significant improvement in mother’s knowledge, attitude and practice regarding diarrhoea etiology and sanitation and hygiene.

Owasis and colleagues [31] conducted a randomized controlled trial in Karachi, Pakistan with mothers who had children less than or equal to 6 weeks of age. The intervention (n=183) consisted of DPT-3/HepB immunization promotion via a 5 -minute presentation using visual displays by a CHW. Pictorial aids addressing the importance of DPT-3/HepB, logistical information on the immunization clinics, and the importance of retaining immunization cards were then left with the mothers. The control group of 183 mothers received a 10–15 minute verbal presentation on general health education, which included basic information on vaccines. After adjusting for immunization status at time of enrollment, which was significantly associated with outcome, the intervention group’s full immunization rate was 32% higher than that of the control four months post intervention.

In southwest Uganda, Brenner et al. [33] conducted a controlled before and after study to assess the impact of CHWs in child under-5 household health promotion following the IMCI strategy. Post-intervention data showed a statistically significant decrease in diarrhoea prevalence of 10.2% and 5.8% in malaria and/or fever in the intervention group, consisting of 606 households. The control group (n=486), which received no household intervention, had non-significant results. As reported by CHWs, after 18 months of the intervention, a decline in underweight children of 5.1% was seen as well as a reduction of under-5 mortality of 53 per cent.

#### Breastfeeding interventions

Five articles, comprising seven different studies relating to breastfeeding, were identified. All interventions involved CHWs promoting approved breastfeeding practices to mothers in their homes, though they differed on timing and intensity of visits and outcomes assessed (Table 2). Of these seven studies, six were randomized controlled trials.

**Table 2:** Visit timing, EBF and diarrhoea rates for cRCT and RCT breastfeeding interventions

**Study**	**2nd Trimester**	**3rd Trimester**	**Days 1-3**	Days 4-5**	**Days 6-7**	**Week 2**	**Week 3**	**Week 4**	**Weeks 5-8**	**Weeks 9-12**	**Weeks 13-16**	**Weeks 17-20**	**Weeks 21-24**	EBF rates***	**Diarrhea incidence**
**Asrasda**				x		x	x		x	x	x	x	x	32% vs. 0%	15% vs. 30.5%
**Haider**		xx	x		x		x		xx	xx	xx	xx	xx	70% vs. 6%	NR
**Morrow 1**	x	x		x		x	x		x					50% vs. 12%	NR
**Morrow 2**		x		x		x								38% vs. 12%	NR
Tylleskar 1*		x		x		x		x	x		x	x		71% vs. 9%	NR
Tylleskar 2*		x		x				x	x	x				51% vs. 11%	NR
Tyllesker 3*		x		x				x	x	x				2% vs. <1%	NR

* Tylleskar 1 is study site Burkina Faso, Tylleskar 2 is study site Uganda and Tylleskar 3 is study site South Africa.

** For studies that indicated visit within the first week, days 4–5 were assigned.

*** EBF rates for Intervention vs. Control at 6 months for all studies except Haider (5 months) and Tylleskar (24 weeks).

NR = not reported.

In Manila, Philippines Agrasada et al. [38] recruited first time mothers who recently gave birth to Low Birth Weight (LBW) babies in hospital for a 3-armed randomized controlled trial. In the first intervention arm CHWs with personal breastfeeding experience educated women (n=68) on exclusive breastfeeding (EBF) and aided in prevention and management of common breastfeeding problems once between days 3–5 and 7–10, and on day 21 and 1.5 months, then once monthly until 5.5 months. In the second intervention, peer counselors using the same visiting schedule as the EBF arm educated 67 mothers on basic child-care practices with some breastfeeding attention. The control (n=69) arm received no household visits. Physicians collected data at hospitals during 7 scheduled appointments for all study arms. Women in the EBF arm were 6.3 times more likely to practice exclusive breastfeeding from two weeks to six months than participants from the basic childhood health intervention and control, with EBF rates of 32%, 3% and 0%, respectfully. Complementary feeding at six months was also significantly higher in the EBF arm, compared to the basic child health arm and control arm (63.2% vs. 31.1% vs. 29.0%). At six months, there was no significant difference in child weight for age between intervention groups, however diarrhoea rates were significantly lower in the EBF arm compared to basic child heath and control groups, 15% vs. 28.3% vs. 30.5%, respectively.

Haider et al. [23] conducted a RCT in the city Dhaka of Bangladesh, to promote EBF through 15 home visits by paid peer counselors with personal breastfeeding experience. Women (n=363) were assigned to both the intervention group and the control group (who received no household visits). In the intervention arm, two visits occurred in the last trimester of pregnancy, three within the early postpartum period and then every two weeks from months 2 to 5. Visits lasted from 20 to 40 minutes and included topics of EBF for 5 months including early newborn holding, and initiation of feeding and discouragement of pre-lacteal and post-lacteal feeding. All measured breastfeeding practices were significantly more prevalent in the intervention group compared to the control with: first hour initiation; 64% vs. 15%, feeding pre-lacteal; 31% vs. 89%, feeding post-lacteals; 23% vs. 47%; EBF during first four days, 56% vs. 9%; EBF on day four, 84% vs. 30%; and EBF to five months, 70% vs. 6 percent.

In a peri-urban area of Mexico, San Pedro Martir, Morrow and colleagues [37] used a 3-arm cRCT with pregnant women and their influential family members. In the first intervention arm participants (n=44) received six home visits from mid pregnancy to eight weeks postpartum by peer counselors to promote healthy breastfeeding practices. In the second intervention arm, 52 women received three household visits, one in late pregnancy and one in the first and one in the second week postpartum. The control population (n=34) received no home visits though all study arms were encouraged to seek standard pregnancy and child facility care. Significant differences in EBF at 2 weeks and 3 months were seen between intervention arms compared to the control, as well as between the two control arms with the 6 visit arm having higher rates than the 3 visit arm. At 2 weeks and 3 months, rates were 80.0% and 67% for 6 visits, 62% and 50% for 3 visits, and 24% and 12% for the control. The intervention had no significant effect on duration of any breastfeeding greater than three months or six months or on diarrhoea incidence in infants between birth and three months.

Tylleskar et al. [22] used the same study design and intervention across three countries, Burkina Faso, Uganda and South Africa. At all sites, a cRCT was arranged using CHWs with similar training and supervision schedules to conduct a behavioural intervention to promote EBF for six months and provide breastfeeding support and education. Women at least seven months or visibly pregnant were recruited and assigned to either the intervention arm consisting of home visits, or the control arm in which women only received existing regular health services. Data was collected based on 24 hour and 7-day recall from the participants.

In Banfora, a rural area in southwest Burkina Faso, 392 women received seven household visits, one in the third trimester, then one in weeks 1,2,4,8,16 and 20 post-partum. Exclusive breastfeeding rates at 12 weeks for intervention and control (n=402) using 7-day recall were significant at 77% and 23%, respectively. At time 24 weeks, EBF rates were reported as 71% for the intervention and 9% for control. At both 12 weeks and 24 weeks there was no significant difference in prevalence of infant diarrhoea for intervention and control arms.

The study in Uganda took place in rural Mbale District, with 396 women in the intervention and 369 in the control cluster. Five household visits by community health workers took place, one in late pregnancy and one each in weeks 1,4,7 and 10 post-partum. Using 7-day recall, EBF at 12 weeks was significantly higher in the intervention than control, with rates of 77% and 34%, respectively. At 24 weeks, 51% of intervention participants compared to 11% of control participants practiced exclusive breastfeeding. As with the study in Burkina Faso, there was no significant difference in infant diarrhoea rates between clusters.

In two peri-urban regions of South Africa, Paarl and Umlazi, and one rural region, Rietvlei, women in the intervention cluster (n=535) received the same home visit schedule as that in Uganda, with 5 visits between the third trimester and week ten. However, in the control cluster peer counselors conforming to the same schedule as that of the intervention, conducted visits to assist mothers in obtaining birth certificates and government grants. Differences in breastfeeding rates between intervention and control were significant at both 12 weeks and 24 weeks (8% vs. 4% and 2% vs. <1%, respectively), though they were remarkably lower than those in the other study sites. The difference in prevalence of infant diarrhoea between clusters at was not significant.

The final study on breastfeeding was a cross-sectional analysis of the intervention arm of a cRCT in rural Sylhet, Bangladesh by Mannan et al. [24]. This study sought to examine breastfeeding problem rates associated with different intervention timings, mostly early visits (within 3 days) compared to non-early visits. Home visits were to be made between postpartum days 1–3, 4–5 and 6–7 to promote newborn care and breastfeeding education and assistance. Only 6% of women who received early CHW visits (between days 1–3) had difficulty breastfeeding compared to 34% of women receiving late visits having difficulties. Pre-lacteal feeding was 2.9 times more common, and the likelihood of having breastfeeding problems was 7.7 times higher in women who did not receive an early visit.

#### Newborn care interventions

Five of the included studies target birth and newborn care preparedness (BNCP) and/or newborn care practices. Bari and colleagues’ [25] intervention aimed to increase appropriate newborn care seeking practices in Tangail, Bangladesh. This cRCT had pregnant women and their families receiving two BNCP home visits at 3 and 8 months prenatal, and postnatal visits on days 0,3,6 and 9 for newborn care education and potential referrals. Controls received no home visits. In the intervention group newborn care seeking from qualified professionals increased significantly compared to the control group, with the intervention group increasing from 31.2% to 60.4% compared to the control group increase from 29.6% to 33.9%. Hospital care also increased significantly and care from an unqualified professionals decreased significantly from 66.7% to 36.7% for the intervention group compared to a minimal reduction from 67.9% to 65% in the control group.

The remainder of the newborn care interventions promote essential newborn care (ENC), specifically the use of skin-to-skin (STS) care to prevent hypothermia and other neonatal morbidities. Two of these studies examined the acceptability and trends in STS care by mothers after a household promotion by community health workers.

In rural Uttar Pradesh, India, Darmstadt et al. [29] examine the acceptability of STS by conducting a before and after study nested within a 3-arm RCT promoting essential newborn care (ENC) practices. Two intervention groups received the same home visits, but differed in that the intervention used a device for measuring body temperature, whereas the control group only had the usual government health services offered to all. Volunteer CHWs, made both antenatal and postnatal home visits to pregnant women and influential family members. Acceptability of STSC taught for home deliveries was high in the two intervention groups with 74.5% of Normal Birth Weight (NBW) and 76% of Low Birth Weight (LBW) receiving the care from their mothers.

Similar to Darmstadt et al., Quasem and colleagues [26] did a case series study on a pilot programme in Sylhet, northeast Bangladesh, for Community Kangaroo Mother Care (CKMC). Essential newborn care and KMC was taught to women seven months pregnant to newly (within 7 days) postpartum using demonstrations and visual aids. One month post intervention women were surveyed for their experiences and findings indicated that 77% initiated KMC, with 85% of LBW babies and 73% of non-LBW babies being given the care. More female neonates than males were exposed to STSC (83% vs. 74%, respectfully). Also mothers providing STSC had more positive exclusive breastfeeding practices and delayed the common, unadvised, practice of immersion bathing.

In rural Uttar Pradesh, India, Kumar et al. [30] conducted a 3-arm cRCT using CHWs to deliver BCC to expectant women and individuals that may have an influence on their health care, argeting positive ENC practices. The two intervention arms received two antenatal and two postnatal home visits from CHWs with one arm utilizing a hypothermia-indicating tool. Neonatal mortality was significantly reduced in both intervention arms compared to the control arm, with a 54% reduction in the ENC only arm, and a 52% in the ENC plus hypothermia measuring tool arm. The intervention had no effect on use of ANC, place of delivery, use of a skilled attendant and immediate umbilical cord care (tying and cutting within thirty minutes), but positively influenced breastfeeding practices with both intervention arms having significantly higher rates of feeding within first hour and lower pre-lacteal feeding. All birth preparedness indicators were significantly enhanced in both intervention arms, with the exception of the pre-identification of a birth attendant. Three newborn thermal practices were significantly more positive in the intervention arms than control: Skin-to-skin (84.9% and 85.5% vs. 10%), bathing within the first day (18.3% and 20.6% vs. 68.1%) and the clothing of baby during massage (5.6% and 5.9% vs. 2.4%).

Similar to Kumar et al. [30] Sloan and colleagues [27] used a BCC intervention to promote CKMC and ENC to expectant and postpartum women and their families in Dhaka and Sylhet, Bangladesh. Using a cRCT, CHWs delivered the programme to the intervention cluster and were responsible for weighing infants during specified visits. Weight measurements within 7 days of birth were collected for 59.0% of the intervention group and 54.2% of the control group. Results from the intervention group show 77.4% ever practicing KMC, with a significantly higher rate for those with home deliveries vs., elsewhere, 85.9% vs. 59.9%, respectively. Women in the intervention group breastfed on average 3.4 hours earlier than those in the control, and 29.3% practiced immersion bathing compared to 72.3% in the control. Infant diarrhoea was significantly reduced in the intervention group (43.6% vs. 39.3%); however, no growth or mortality differences were observed.

#### Mother psychosocial well-being interventions

Two studies were identified that used CHWs to provide social support or therapy to pregnant women to affect positive health outcomes for both mother and child.

Cooper and colleagues [34] identified women in their last trimester of pregnancy in Khayelitsha, South Africa for a RCT to provide support and guidance for parenting. Two hundred and twenty women in the intervention group received 16 home visits (2 antenatal visits, weekly visits for 8 weeks postpartum, followed by bi-weekly visits for two months) lasting approximately an hour each, for promotion of sensitive and responsive interactions with infants. Women in the control group (n=229) received their normal local services with no additional home visits. Women in the intervention group had significantly more sensitive and less intrusive mother-infant interactions at both 6 and 12 months. Secure infant attachment was also significantly higher in the intervention (74%) than the control (63%). Though maternal *depressed mood* at 6 months was lower in the intervention group there was no effect on maternal *depressive disorder*.

In rural Pakistan, Rahman et al. [32] conducted a cRCT with women in their third trimester of pregnancy with perinatal depression following DSM-IV criteria. The “Thinking Healthy Programme”, a cognitive behavioural therapy, was used by CHWs during 4 visits in the last month of pregnancy, 3 in first month postnatal followed by 1 session per month for the succeeding 9 months. A CHW visited the women in control clusters following the same schedule but without administering the Thinking Healthy Programme. Outcomes were measured at 6 and 12 months for both mother and child. At both 6 and 12 months maternal depression was significantly lower in intervention clusters compared to control clusters, 23% vs. 53%, and 27% vs. 59%, respectively. Also, disability function scores and perceived social support were all significantly improved in the intervention clusters. However, there were no significant differences in child weight-for-age or height-for-age at 6 and 12 months between groups.

## Discussion

This review, consistent with previous systematic reviews for MCH [10,11,15], found that there is a paucity of research from LMICs, especially in sub-Saharan Africa where the highest levels of both maternal and child mortality occur [42]. By allowing a greater variety of study designs to be included, as opposed to the traditional systematic review criteria of only randomized controlled trials, a larger number of studies were identified for inclusion in this review. The same scope of CHW’s involvement in MCH prevention interventions identified in other reviews [11,14,15,43,44] was not identified here partially because of the often multidimensional role of a CHW, consisting of both prevention and curative activities, which excluded studies as this review was interested in exclusively preventive activities. This finding in itself warrants further investigation into CHW performance, motivation and retention – main obstacles facing CHW programmes – as there is debate whether single interventions or multifaceted interventions are best suited for CHWs [7,45].

The operational definitions included in this review may have limited the results for some prevention interventions previously identified as being within the scope of a CHWs’ ability [43]. The definition of CHWs chosen [39] “…members of the communities where they work, should be selected by the communities, should be answerable to the communities for their activities, should be supported by the health system but not necessarily a part of its organization, and have shorter training than professional workers” was difficult to operationalise in search terms and its comprehensive nature may have excluded community workers meeting some but not all of these criteria. We recognise that several studies of effective interventions by community members may have been excluded because of this. For example, a study assessing CHWs’ effectiveness in delivering misoprostol to prevent postpartum hemorrhage in Afghanistan [46], that may have been suitable for this review, was not identified during the search due to title search limitations. Further research in this area with a more expansive search strategy to encompass prevention techniques that may not have been within the scope of this review is recommended to add to the existing literature.

Though an in-depth analysis of the best practices and main problems surrounding CHW programmes is not the purpose of this review, it is important to recognise the influencing factors for a successful strategy as identified throughout the literature. Community participation and ownership, leadership and adequate resources, appropriate selection, training, continual learning, support and supervision, as well as incentives to influence retention and motivation, are all essential considerations when designing and implementing CHW programmes [7,11,14,43], though this list is not exhaustive. Each identified study’s success or lack of, may be influenced by the aforementioned factors that can vary between contexts and should always be considered when investigating and initiating new programmes.

### Narrative synthesis

#### Malarial interventions

Moderate quality studies assessed CHWs delivering preventive treatment for malaria, which is responsible for 7% of child under-5 deaths and affects up to 50% of women during pregnancy [47]. Community health workers were effective in delivering the IPT medication to targeted households as well as positively influencing the malaria prevention technique of sleeping under INTs. These two studies present evidence for the capacity of CHWs to be trained to deliver Intermittent Preventative Treatment for both children under-5 and women during pregnancy; however, such strategies should involve high levels of training and supervision and require strong government/funder commitment to ensure drug supply. These types of programmes should have regular supervisory visits, frequent auditing of resources, steady supplies, community promotion policies and education, and strict and enforced regulations [48]. There is a need for further studies to assess the effectiveness of CHWs delivering interventions on malarial prevention in a wider range of contexts, over longer periods of time, and with varying support structures in place.

#### Health education interventions

Overall, moderate quality evidence showed the effectiveness of CHWs in health education interventions. All three included studies reported significant results, though due to differences in interventions a cross-comparison of results was not possible. In two different interventions [28,31] CHWs were able to promote messages on both food safety and immunizations in order to increase appropriate practices, and were effective in decreasing diarrhoea and increasing immunization rates, respectively. Both of these interventions consisted of simple, targeted messages and utilized visual aides. In a more multifaceted intervention by Brenner et al. [33] CHWs were associated with a decrease in diarrhoea, malaria, underweight prevalence and mortality in children under-5 through household education on various child health issues.

Although this review does provide support for the use of CHWs in the delivery of health education interventions on food safety, immunizations and child under-5 care, more directly comparable interventions are needed before any conclusive evidence of effectiveness can be presented.

#### Breastfeeding interventions

In the developing world, less than 40% of infants are exclusively breastfed until 6 months of age, a practice that prevents diarrhoea and acute respiratory illness, as well as facilitates health growth and development, strengthens the immune system and provides essential vitamins and minerals [49]. Strong quality of evidence from this review indicates the effectiveness of CHWs in promoting positive breastfeeding practices. The evidence that this increase in breastfeeding has the desired effect on reducing diarrhoea in infants is not as available.

Although all interventions increased EBF rates significantly, there appears to be a correlation between rate increase and the timing and intensity of visits, though a statistical analysis to confirm this correlation is beyond the scope of this review. The two interventions with the highest EBF rate difference between intervention and control had the highest and third highest visit numbers, with a 61% difference and 15 visits and a 62% difference with 7 visits [22,23]. However, the study with the second highest visit frequency [38] did not have a prenatal visit which may contribute to its lower difference rate. Considering Mannan et al. [24] findings and the evidence from the RCTs and cRCTs, four characteristics for minimum visit schedules should be considered when designing an intervention: 1) at least one prenatal visit; 2) one early (days 1–3) visit; 3) continue visits past first month postnatal; and 4) a higher frequency of visits can increase success, though a threshold may be reached at 7 visits.

Only one study [38] reported a significant difference in infant diarrhoea prevalence, which may be attributed to the population’s characteristics, as these women were the only group recruited from a hospital and continued to return for data collection. Also, as EBF data is typically self reported, problems with recall or a misunderstanding of the definition of EBF may have lead to some women false-reporting EBF, while in actuality complementary feeding occurred. Unhygienic and unsanitary practices may have also contributed to a lack of diarrhoea reduction, such as failure to wash hands before a feeding or after dealing with defecation.

Training, workload and supervision for CHWs in these interventions varied, though a majority of interventions required that all CHWs had personal experience with breastfeeding. Overall, this review shows quality evidence for the effectiveness of CHWs in increasing EBF rates, but does not support their ability to decrease diarrhoea prevalence in infants through breastfeeding promotion.

The suggested minimal characteristics for CHW programmes to promote appropriate breastfeeding should be further explored, and with more supporting evidence be promoted universally to achieve higher success. However, the main priority for appropriate breastfeeding research should be to investigate the relationship between EBF and diarrhoea rates, appropriate EBF practices and reporting, and hygiene promotion in combination with breastfeeding interventions, as the findings in this review are very worrisome.

#### Newborn care interventions

Forty percent of all under-five deaths occur in the first 28 days of life, the neonatal phase [6]. Though simple techniques and strategies are known to reduce the burden of mortality in this age group, lack of knowledge and resources inhibit success in many areas. This review found moderate to strong quality evidence for the use of CHWs in promoting essential newborn care strategies.

Community health workers were effective in increasing appropriate health seeking behaviours for newborns by educating mothers on the importance of qualified care. They were also effective in promoting the use of skin-to-skin care for all newborns regardless of birthing location. Post intervention, acceptability of STS practice by mothers was high, though the practice was not utilized to the extent initially taught by the CHWs, with women stopping earlier or doing less hours per day than recommended. Low birth weight babies, girls and neonates born within the household were more likely to receive STS care from their mothers.

In Kumar et al. [30], CHWs were able to reduce neonatal mortality, increase birth preparedness indicators and breastfeeding practices and increase appropriate newborn thermal practices. Sloan et al. [27], however, using a similar multifaceted intervention did not observe significant changes in mortality or growth, but were able to reduce diarrhoea and harmful practice such as immersion bathing, and increase appropriate breastfeeding practices. Reasons for the difference in effectiveness for certain indicators, especially neonatal mortality, are unknown and should be further explored. The evidence from this review does support the use of community health workers in delivering newborn care interventions to prevent morbidity and mortality, especially the use of STS care, though further studies are needed to assess the most effective components of the interventions.

#### Maternal psychosocial interventions

Moderate quality evidence was reported for CHWs providing psychosocial interventions for women. Maternal depression and poor parent–child interaction and attachment have both been found to increase developmental and growth problems, and ill health in children. In Cooper et al. [34] through home sessions providing support and guidance for mothers, CHWs were able to increase positive interactions and healthy attachment behaviours, though they had no effect on maternal depression rates. Rahman et al. [32], however, found CHWs effective in helping women recover from maternal depression. The difference in success for maternal depression between the two studies may be accounted for by two factors: firstly, women in Rahman were prediagnosed with depression, therefore CHWs were not used to prevent but to help rehabilitate mothers and secondly, Rahman et al. [32] focused more intensively on targeting the depressive disorder whereas Cooper et al. [34] employed a more general parenting intervention.

Perinatal and postpartum depression are greatly under researched in LMIC and are estimated to be three times more prevalent than in high income countries, with a range in prevalence from 10-41% [50], though one study found rates up to 60% [51]. With an already severe HRH crisis crippling even basic health care services in most LICs, mental health services are essentially nonexistent in many areas, as evidence by the fact that LICs on average have 0.05 psychiatrists and 0.16 psychiatric nurses for every 100,000 people, with these rates being 200 times greater in high-income countries [52]. Community based strategies and CHWs may present a means to provide mental health services for individuals, especially women suffering maternal depression and associated disorders. More research into the capacity of CHWs to deliver interventions for maternal mental health in LMICs is highly enouraged and needed to identify feasibility and best practices as to reduce negative health outcomes for both mother and child.

In this review, both interventions provide evidence for the use of CHWs in delivering psychosocial interventions to mothers, however the logistics of these interventions must be thoroughly considered before being implemented on a larger scale or in different settings as both required intense supervision and training and high frequency of visits, 16 each. Interventions should also have a specific focus and not attempt to over-strain the capacity of the community health workers.

Overall, the studies in this review showed potential for CHWs to deliver exclusively preventive interventions for MCH in low-and-middle income countries. Several main characteristics did emerge: CHWs often had high and intense levels of training and supervision; support services were well defined and strong; and home visits were often quite frequent. Though these are all previously identified characteristics of strong CHW programmes, it brings into question the feasibility of scaling-up such programmes.

### Limitations

Two major limitations of this study are the lack of a second reviewer for each of the stages and restricted time. Though efforts were taken to maintain rigor and thoroughness throughout the review, the time limitation may have also influenced the analysis and interpretation of results. Publication bias, the notion that studies with positive results are more likely to be published, may have lead to some conducted studies with negative results not being published and therefore being less likely to be identified through the search strategy. Bias may have also been introduced in the search terminology used. Efforts were taken to construct the search terms to be inclusive of all possible definitions and vocabulary; however, it is possible that certain terms were excluded and may have influenced the returned results. It is also recognised that since this review aimed to identify studies with CHWs preforming prevention only activities, other sources of evidence for their effectiveness in this area may have been excluded, especially within multiple intervention programmes. Other authors, for example Perry and Zulliger [43], have compiled a more broad list of CHW programmes for further reading.

## Conclusions

This review found moderate evidence that community health workers are effective in delivering preventive interventions for maternal and child health in low- and middle-income countries. Further investigation into CHWs delivering preventive interventions should be conducted to strengthen support for this role, as well as the practically of scaling-up such initiatives under similar programme guidelines.

Evidence from this review suggests several strategies that should be further explored, including combining hygiene education with breastfeeding interventions with the prospect of reducing diarrhoea rates in infants, using visual aids, which can be left with the mother as educational tools, and specifically targeting health messages. Variations in interventions, training and outcomes make it difficult to compare all included studies, however some important findings emerged from this research:

1. Community health workers are effective at increasing acceptability of mother-performed practices, such as skin-to-skin care and exclusive breastfeeding.

2. Community health workers are capable of providing interventions beyond their traditional scope and with more intense training, such as those of a psychosocial nature or delivering scheduled intermittent preventive treatment for malaria. Further research into CHWs providing services for mental health issues is highly encouraged to provide services for these imperative, yet vastly under resourced, issues.

3. Community health workers are effective in delivering health promotion or education, especially with simple, targeted messages. The use of visual aides may also be very valuable in relaying these messages.

It is recommended that policy makers explore the option of increasing CHW’s responsibility in the prevention of maternal and child morbidity and mortality, though interventions need to be tailored to specific settings and contexts. Prevention services provided by CHWs may serve as a tactic against the HRH crisis, be cost effective in both training and provision due to lesser responsibility compared to curative interventions and may reach more households as many interventions are educational promotions and thus allow for knowledge transfer between households.

## Abbreviations

ANC: Antenatal Care; BCC: Behaviour change communication; BNCP: Birth and newbown care preparedness; CHW: Community health worker; CKMC: Community Kangaroo mother care; cRCT: Cluster randomized controlled trial; EBF: Exclusive breastfeeding; ENC: Essential newborn care; EPHPP: Effective public health practice project; EPPI: Evidence for policy and practice information; HRH: Human resources for health; IPT: Intermittent preventative treatment; ITN: Insecticide treated net; KMC: Kangaroo mother care; LBW: Low birth weight; LMIC: Low – and middle-income countries; MCH: Maternal and child health; MDG: Millennium development goal; NBW: Normal birth weight; STS: Skin to skin; RCT: Randomized controlled trial; WHO: World health organization.

## Competing interests

The authors declared that they have no competing interests. There were no sources of funding for this review.

## Authors’ contributions

BG performed the literature search, screened articles for inclusion and analysed and interpreted the data, with EM consulting and reviewing BG’s work. BG and EM reviewed all full text screened articles and together selected the included studies. BS drafted the manuscript. EM reviewed, edited and revised the manuscript. Both authors conceived and designed the study and approved the final version.

## Pre-publication history

The pre-publication history for this paper can be accessed here:

http://www.biomedcentral.com/1471-2458/13/847/prepub

## Supplementary Material

Additional file 1 Electronic search strategy. Click here for file

Additional file 2 **Additional names for community health workers.** Compilation of different names used worldwide for community health workers, which were used in the search strategy. Click here for file

Additional file 3 **Inclusion and exclusion criteria table.** Detailed list of criteria for the purpose of this review. Click here for file

Additional file 4 Data extraction tool and included studies’ characteristics. Click here for file

Additional file 5 **Search log.** Dates searched and returned results for the databases used in this review. Click here for file

Additional file 6 **Excluded full text reviewed characteristics.** Additional information on the studies that had their full texts reviewed, including the reason they were excluded from this review. Click here for file

Additional file 7 **CHW characteristics.** Characteristics of the community health workers for each of the included, including training, supervision, and other factors that should be considered when analysing CHW programmes. Click here for file
