# Supplementary material for: Effectiveness of community health workers delivering preventive interventions for maternal and child health in low- and middle-income countries: a systematic review
Source: BMC Public Health. 2013 Sep 13;13:847. doi: 10.1186/1471-2458-13-847 (PMC3848754; doi:10.1186/1471-2458-13-847)
Supplement: Additional file 1 — Electronic search strategy. [file 1471-2458-13-847-S1.docx]

**Additional file 1 – Electronic search strategy**

1. “CHWs”:ti

2. “LHWs”:ti

3. “VHWs”:ti

4. “Community Health Worker”:ti or “Community Health Workers”:ti

5. “Voluntary Worker”:ti “Voluntary Workers”:ti

6. “Health Auxiliary”:ti

7. “Health Visitor”:ti Or “Health Visitors”:ti

8. ‘Health extension worker’:ti:ab OR ‘Health extension workers’:ti:ab

9. ‘Lady health worker’:ti:ab OR ‘Lady health workers’:ti:ab

10. (Lay or voluntary or volunteer* or untrained or unlicensed or nonprofessional* or non professional* adj1 worker* or visitor* or attendant* or aide or aides or support* or person* or helper* or carer* or caregiver* or consultant* or assistant* or staff or visit* or midwife or midwives):ti AND (maternal or women* or child* or infant* or neonatal* or MCHN or MCH or ‘maternal and child health and nutrition’ or ‘maternal and child health’):ti AND (prevent* or deterren* or education or teach* or knowledge or literacy or precaution* or promot* or ‘knowledge passing’ or counsel* or advise or ‘preventive therapy’ or outreach or encourage* or ‘health promotion’ or ‘health education’ or ‘disease prevention’ or ‘illness prevention’):ti

11. ‘Lay volunteer’:ti:ab

12. ((#1-9) and (maternal or women* or child* or infant* or neonatal*or MCHN or MCH or ‘maternal and child health and nutrition’ or ‘maternal and child health’)):ti:ab

13. (“allied health personnel” or “allied health worker” or “support worker” or “home health aide”):ti and (maternal or women* or child* or infant* or neonatal* or MCHN or MCH or ‘maternal and child health and nutrition’ or ‘maternal and child health’):ti:ab

14. (“Trained” or “unlicensed” adj1 volunteer* or ‘health worker’ or mother* or ‘community member’):ti AND (maternal or child* adj1 health or nutrition):ti:ab

15.((“Community” or “health”) adj3 (“volunteer” or “aide” or “support” or “extension” or “assistant” or “surveyor” or “distributor” or “auxiliary”)):ti AND (maternal or women* or child* or infant* or neonatal* or MCHN or MCH or ‘maternal and child health and nutrition’ or ‘maternal and child health’):ti:ab

16. (“Linkworker” or “link worker”) and (maternal or women* or child* or infant* or neonatal* or MCHN or MCH or ‘maternal and child health and nutrition’ or ‘maternal and child health’):ti:ab

17. ‘Barefoot doctor’:ti:ab

18. (Outreach):ti and (maternal or women* or child* or infant* or neonatal*or MCHN or MCH or ‘maternal and child health and nutrition’ or ‘maternal and child health’):ti:ab

19. (Care or aide* or support or intervention* or visit* or education or teach* or prevent*) adj3 (lay or volunteer* or voluntary or community or “community worker*” or “community healthcare worker” or “community health care worker”):ti:ab

20. “Community health worker” and “prevention”

21. “Community health worker” and “under 5”

22. “Community health worker” and (maternal or child) And (developing or “low-income” or “resource poor” or “global south” or “third world”)

23. “Activista” or “Agente comunitario de salud” or “Agente comunitario de saude” or “Anganwadi” or “Animatrice” or “Barangay health worker” or “Basic health worker” or “Brigadista” or “Colaborador voluntario” or “Community drug distributor” or “Community health agent” or “Community nutrition worker”or “Community resource person” or “Monitora” or “Mother coordinator” or “Outreach educator” or “Promotora” or “Rural health motivator” or “Shastho shebika” or “Shastho karmis” or “Sevika” or “Village drug-kit manager” or “Village health helper”
