# Supplementary material for: Effectiveness of community health workers delivering preventive interventions for maternal and child health in low- and middle-income countries: a systematic review
Source: BMC Public Health. 2013 Sep 13;13:847. doi: 10.1186/1471-2458-13-847 (PMC3848754; doi:10.1186/1471-2458-13-847)
Supplement: Additional file 2 — Additional names for community health workers. Compilation of different names used worldwide for community health workers, which were used in the search strategy. [file 1471-2458-13-847-S2.docx]

### Additional file 1 – Additional names for community health workers

| Activista  Agente comunitario de salud  Agente comunitário de saúde  Allied health personnel  Anganwadi  Animatrice  Auxiliary health worker  Barangay health worker  Barefoot doctor  Basic health worker  Brigadista  Colaborador voluntario  Community assistants  Community drug distributor Community health agent  Community Health Aides Community health promoter  Community health  representative | Community health volunteer  Community health worker  Community mobilizer  Community nutrition worker Community resource person  Community support worker  Community volunteers  Female community health  volunteer  Female multipurpose health  worker  Health and nutrition worker  Health promoter  Home health aides  Kader  Lady health worker  Lay Health Worker | Link worker  Maternal and child health  worker  Monitora  Mother coordinator  Outreach educator  Paramedical worker  Promotora  Peer volunteer  Rural health motivator  Rural Health Worker  Shastho shebika  Shastho karmis  Sevika  Village drug-kit manager  Village health helper  Village health worker  Voluntary workers |
| --- | --- | --- |
