# Supplementary material for: Effectiveness of community health workers delivering preventive interventions for maternal and child health in low- and middle-income countries: a systematic review
Source: BMC Public Health. 2013 Sep 13;13:847. doi: 10.1186/1471-2458-13-847 (PMC3848754; doi:10.1186/1471-2458-13-847)
Supplement: Additional file 3 — Inclusion and exclusion criteria table. Detailed list of criteria for the purpose of this review. [file 1471-2458-13-847-S3.docx]

### Additional file 2 – Inclusion and exclusion criteria table

|  | **Inclusion** | **Exclusion** |
| --- | --- | --- |
| **Target Beneficiaries** | Pregnant women to 42 days post termination  Children under 5  Caregivers of children under 5 | Non-distinguishable groups |
| **Interventions** | Preventative  Maternal and Child Health  Household level  Delivered by CHW | Mixed intervention where inclusion criteria cannot be distinguished in administration and outcomes  CHWs not clearly defined |
| **Outcomes** | All included | ---- |
| **Study Design** | Experimental and Observational | Low quality/high bias assessed by reviewer |
| **Setting** | Low and Middle Income Countries (LMIC) | Not LMIC at time of study initiation |
| **Time Period** | Published 1990 and onwards | Published pre-1990 |
| **Language** | All languages | English abstract not available and/or no translator available |
| **Publication Type** | Primary research | Not a primary research report |
