# Supplementary material for: Effectiveness of community health workers delivering preventive interventions for maternal and child health in low- and middle-income countries: a systematic review
Source: BMC Public Health. 2013 Sep 13;13:847. doi: 10.1186/1471-2458-13-847 (PMC3848754; doi:10.1186/1471-2458-13-847)
Supplement: Additional file 4 — Data extraction tool and included studies’ characteristics. [file 1471-2458-13-847-S4.docx]

| Additional file 3 – Data extraction tool and included studies’ characteristics | | | | | | | | | | |
| --- | --- | --- | --- | --- | --- | --- | --- | --- | --- | --- |
| **Author and Year** | **Setting** | **Study Design** | **Population** | | **Intervention** | | **Group Allocation Method** | **Outcomes^[[1]](#footnote-1)^** | | **Quality^[[2]](#footnote-2)^** |
|  |  |  | **Intervention** | **Study** | **Intervention Group** | **Control/**  **Comparison** |  |  |  |  |
| **Interventions Targeting Malaria** | | | | | | | | | | |
| **Ahorlu 2009** | Volta region, Ghana | Before and After | Children age 6-60 months | Children in intervention and their caretakers | IPTc delivered by community assistants every 4 months for 1 year, consisting of 10mg/kg body weight of amodiaquine daily and 4mg/kg of artesunate daily, over 3 days. 357 in intervention | Baseline malaria parasite prevalence from 174 children. All caretakers of selected children interviewed.  One year after initiation, evaluation of 80% of children for parasite prevalence | All caretakers interviewed at baseline.  Children randomly selection for parasite prevalence | Parasite prevalence C1 vs. I1: 25% vs. 3%, (sig difference of over 8-% p <0.00, Mann-Whitney)  Anemia, significant (p 0.004 Mann-Whitney test) decrease from C1vs I1: 27.6% vs. 16.8%  Bed-net usage C1 vs. C2: 98% vs. 100%  Insecticide treated bed-net use C1vs.C2: 38.5% vs. 60% | | ++/ |
| Okeibunor  **2011** | Akwa Ibom Nigeria | Before and After parallel group design | All pregnant women residing in programme areas | Women who had given birth within 6 months | 3 groups received community directed intervention (CDI) for delivery of ITNs and two doses of SP for IPTp and basic counseling services for pregnant women by community directed distributors (CDD) | 3 groups had no CDI.  Both Intervention group and control had increased support for public health facilities (training, resources, supplies) | Non-random group assignments at local government level by for balanced samples. | ITN pregnancy mean change I vs. C: 0.11 vs. 0.03 (p<0.01)  ITN post delivery mean change I vs. C: 0.10 vs. 0.02 (p<0.001)  2+ SP doses mean change I vs. C: 0.57 vs. 0.21 (p<0.001)  ANC mean change I vs. C: 0.22 vs. 0.21, significant change, no effect difference | | ++/ |
| **Interventions for Health Education** | | | | | | | | | | |
| **Sheth 2004** | Baroda urban slums, India | Before and After | Mothers of underprivileged children 6-24 months | Mothers and children included in the intervention | Anganwadi workers educating mothers on Food safety education (FSE) though home visits and educational materials. 3 main messages: proper hand washing, avoid leftovers, clean surroundings. | Baseline survey of 200 households included in intervention, compared to post intervention survey | 8 Anganwadi centers randomly chosen, from these 200 HH purposely selected | | Diarrhea episode C1 vs. I1: 92% vs. 39.5%  Environmental sanitation C1 vs. I1: 50.5% vs. 14% (poor) (p<0.001)  Personal hygiene C1 vs. I1: 38.5% vs. 30.5% (poor)(p<0.001)  Microbial load detected mothers C1 vs. I1: 90% vs. 25%. Children: 100% vs. 90%  Mother’s KAP of diarrhea etiology, sanitation and hygiene all significantly (p<0.001) increased | + |
| Owais 2011 | Karachi, Pakistan | RCT | All mothers in study area having live child less than or equal to 6 weeks old that agreed to participate (n=366) | All mothers included in intervention | DPT-3/HepB immunization promotion using CHWs to give one, 5 minute presentation using pictorial aids addressing: importance of DPT-3/HepB, logistical information and importance of retaining immunisation card. n= 183 | Verbal education session by CHWs, approx. 10-15 min, adapted from curricula developed by Pakistan’s MOH for Lady Health Workers including some general information on vaccines. n=183 | Randomized and stratified at enrollment sites, block randomized at individual level | | After 4-month follow-up, full immunization (3 doses plus vaccination card) for I1 vs. C1: 72.1% vs. 51.7%. I1 increased rates by 39% (95% CI)  Retaining vaccination cards I1 vs. C1 : 81% vs. 69.1%  Immunisation status at enrollment significantly associated with outcome (p<0.05), after adjusting rates in intervention group improved by 32% (95% CI) | +++ |
| Brenner 2011 | South-west, Uganda | Control Before and After | Families with children under-5 residing in intervention area | HH with children under-5 in both intervention and control areas | CHWs mainly for child health promotion based on IMCI health promotion.  CHWs aid in illness management.  CHW identification and reporting of children and health issues. | (C1) Control: No trained CHWs  Comparison: Baseline evaluation vs. post-intervention evaluation of Intervention group vs. Control group. | Randomized at community level. Study evaluation randomized by villages per perish. | | Underweight (z-score >2SD, weight-for-age) change, 95%CI: I1 vs. C1; -5.1% vs. -1.0%, Fever/malaria change, 95%CI, I1vs.C1: -5.8% vs. -3.1%, Diarrhea change, 95%CI, I1vs. C1: -10.2%vs. -2.8%, Mosquito net in HH change, 95%CI, I1vs.C1: 37.2%vs.12.5%  Measles vaccine change, 95%CI, I1vs.C1: 10.6%vs.6.8%, ANC ≥ 4 change, I1vs.C1: 6.9% vs. 6.6%, Under-5 mortality decreased 53.2% in I1 | ++ |
|  |  |  |  | **Intervention** | **to Support** | **Breastfeeding** |  |  | |  |
| Agrasada  2005 | Manila, Philippi-nes | RCT | First-time mothers, 18 or older, vaginal delivery of LBW singleton in hospital, intention to BF, infant born between 37-42 wks gestation | Mothers and their infants enrolled in study | I1: Eight home visits by peer counselor on days age, 3-5, 7-10 and 21, and 1.5 months, then monthly up to 5.5 months, promoting EBF and assisting mothers in prevention and management of BF problems  I2: Visits by peer counselor educating on child care, following same schedule as I1, | No HH intervention. | Individual level by table of random numbers | EBF from 2wk to 6mth 6.3 times (p<0.001) more likely to EBF than I2 and C1.  I1 and C1 difference, p=0.95  EBF to 6 mths, I1vsI2vsC1: 32%vs 3% vs. 0%  Complementary feeding at 6 mths, for I1vs.I2vs.C!: 63.2%vs.31.3%vs.29.0% (p<0.001)  Weight for age difference between infants not significant at 6 months  Diarrhea rates., I1vs.I2vs.C1: 15%vs.28.3%vs.30.5%) | | ++/ |
| Haider 2000 | Dhaka, Banglad-esh | RCT | Pregnant women in third trimester and their key family members | Pregnant women aged 16-35, no more than three living children or parity five. | 15 home visits (2 in last trimester, 3 early postpartum including within 48 hrs. delivery, fortnightly in mths 2-5) by CHW lasting 20-40min. Included benefits of EBF for 5 mths, early initiation, holding baby within minuets, discouraged prelacteal and postlacteal foods. | No CHW HH visits. | Randomized by zone (20 and 20) by random number table | Early holding of infant I1 vs. C1: 1hr vs. 2hr (p<0.0001)  Early BF, median time I1vs. C1: 1hr vs. 9hr.  First hour initiation I1vs. C1: 64% vs. 15% (p<0.0001)  Feeding prelacetal I1vs. C1: 31% vs. 89% (p<0.0001)  Feeding postlacetals I1vs C1: 23% vs. 47% (p<0.001)  EBF first 4 days I1vs. C1: 56% vs. 9% (p<0.0001)  EBF on day 4, I1vs. C1: 84% vs. 30% (p<0.001)  EBF 5 mths, I1vs. C1; 70% vs. 6% (p<0.0001) | | +++ |
| Mannan 2008 | Rural Sylhet, Banglad-esh | Cross-sectional study nested in cRCT | Expectant women and others involved in maternal and/or newborn care/support | Women in study, excluded if: facility delivery, stillbirth or abortion, CHW visit timing problematic, morbidity w/ or w/out feeding problems | CHW made 2 antenatal (second and third trimester) for BNCP and 3 postpartum (between days 1-3, 4-5 and 6-7) for Newborn care. | Intervention: Comparison arm received no CHW newborn care intervention  Study - comparison between individuals in Intervention, timing and occurrence of CHW visit, and newborn feeding problems | 24 clusters randomized into either intervention or control | Feeding problems for early visits vs. late visits: 6% vs.34%  No early visits 7.7 times more likely to have problems  Prelactal feeding 2.9 times more likely if no early visit | | ++ |
| Morrow 1999 | San Pedro Martir, Mexico | cRCT | All identified pregnant women residing in study area | Women included in study and their infants | I1: six home visits to promote BF, 1 mid and 1 late pregnancy, and in week 1, 2, 3, and 8 postpartum.  I2: three home visits to promote BF, 1 late pregnancy and week 1 and 2 postpartum | No HH intervention | Area mapped into 39 clusters, and randomized using computer generated schedule | EBF at 2wks and 3 mths in:  I1: 80% and 67%  I2: 62% and 50%  C1: 24% and 12%  (Between I1+I2 vs. C1, p<0.001, and I1vsI2 p=0.015)  EBF from 2wks-3mths, I1vs.I2vs.C1: 50% vs. 38% vs. 12% (p<0.001)  Duration any BF greater than 3 mths, and 6 mths I1+I2 vs. C1: 95% vs. 85% (p=0.39) and : 87% vs. 76% (p=0.09)  Diarrhea incidence in infants 0-3 mths, I1+I2 vs. C1: 12% vs. 26% (p=0.29) | | ++ |
| Tylleskar 2011 | Rural Banfora,  Burkina Faso | cRCT | Pregnant women, 7 months or visibly pregnant, residing in selected cluster | Mother-infant pairs identified at 3-week postpartum with no multiple births, or disability in mother or child that could interfere with BF | Behavioural intervention trial with peer counselors providing HH breastfeeding support and education, based on WHO courses for BF counseling and encouraged EBF for 6 months. Visit 1 in third trimester, then first week 1, 2, 4, 8, 16 and 20. | Regular health care services | Randomized at community level via computer generated randomized sequence | EBF at 12 wks, based on 24-h and 7-day recall, respectively, I1vs.C1: 79%vs.35% and 77%vs. 23%  EBF at 24 wks, based on 24-h and 7-day recall, respectively, I1vs.C1: 73%vs.22% and 71%vs. 9%  Prevalence of infant diarrhea at 12 weeks and 24 week between I1 and C1 is not significant. | | +++ |
|  | Mbale District, Uganda | cRCT | Pregnant women, 7 months or visibly pregnant, residing in selected cluster | Mother-infant pairs identified at 3-week postpartum with no multiple births, or disability in mother or child that could interfere with BF | Behavioural intervention trial with peer counselors providing HH breastfeeding support and education, based on WHO courses for BF counseling and encouraged EBF for 6 months. Visit 1 in third trimester, then first week 1, 4, 7 and 10. | Regular health care services | Randomized at community level via computer generated randomized sequence | EBF at 12 wks, based on 24-h and 7-day recall, respectively, I1vs.C1: 82%vs.44% and 77%vs. 34%  EBF at 24 wks, based on 24-h and 7-day recall, respectively, I1vs.C1: 59%vs.15% and 51%vs. 11%  Prevalence of infant diarrhea at 12 weeks and 24 week between I1 and C1 is not significant. | | +++ |
|  | Paarl , Umlazi & Rietvlei South Africa | cRCT | Pregnant women, 7 months or visibly pregnant, residing in selected cluster | Mother-infant pairs identified at 3-week postpartum with no multiple births, or disability in mother or child that could interfere with BF | Behavioural intervention trial with peer counselors providing HH breastfeeding support and education, based on WHO courses for BF counseling and encouraged EBF for 6 months. Visit 1 in third trimester, then first week 1, 4, 7 and 10. | Same schedule visits by peer counselors to assist in obtaining birth certificates and government grants | Randomized at community level via computer generated randomized sequence | EBF at 12 wks, based on 24-h and 7-day recall, respectively, I1vs.C1: 10%vs.6% and 8%vs. 4%  EBF at 24 wks, based on 24-h and 7-day recall, respectively, I1vs.C1: 2%vs. <1% and 2%vs. <1%  Prevalence of infant diarrhea at 12 weeks and 24 week between I1 and C1 is not significant. | | +++ |
| Cooper 2009 | Khayelit-sha, South Africa | RCT | Women in last trimester living in study areas | 449 women pregnant within study areas | 220 women from late pregnancy to 6 months postpartum  I1: 2 antenatal visits, weekly visits for 8 weeks postpartum, biweekly for following 2 months (total 16 1-hour sessions in 5 months) by CHW who provided support and guidance in parenting, in addition to normal local services | 229 women received normal local services involving fortnightly CHW visits | Assignment by minimization taking into account location, depression and planned pregnancy | O1: Mother-infant interaction, more sensitive and less intrusive  O2: Secure infant attachment, I vs. C: 74% vs. 63%  03: Depression: Depressive disorder different not significant, Maternal depression significant at 6 months only. | | ++/ |
| Rahman 2008 | rural Rawalpi-ndi, Pakistan | cRCT | Married women age 16-45 with perinatal depression (DSM-IV criteria) in last trimester | Mothers from intervention and control and their infants | “Thinking Healthy Programme”, cognitive behavioural therapy, with 1 session/week for 4 weeks in last month pregnancy, 3 sessions first month postnatal, sessions 1 per month for 9 months after | Equal number of visits and structure, by LHW not using Thinking Healthy Programme | 40 Union clusters randomly assigned by independent trial center, intervention or control | Maternal depression at 6mths I1vs. C1: 23% vs. 53% (p<0.001), and 12 mths 27% vs. 59% (p<0.001).  Disability, function score and perceived social support in I1 all improved significantly.  Weight-for-age (Zscore) at 6mths I1vs.C1: -0.83 vs. -0.86 (p=0.07) and 12 mths: -0.64 vs. -0.8 (p=0.3)  Height for age (Zscore), I1vs.C1 at 6mths: -0.2.03vs. -2.16 (p=0.3) and 12 mths: -1.10 vs. -1.36 (p=0.07) | | +++ |
| Bari  2006 | Tangail district,  Banglad-esh | cRCT | Identified pregnant women and their families | Pregnancy outcome in last 36 mths  Interim - pregnancy outcome last 7-8 mths | CHWs deliver maternal and newborn-care interventions, during visits at 3 and 8 mths for BNCP, and at day 0,3,6 and 9 for newborn care and referrals. Focus on proper health seeking practices | Control: No CHW HH visits.  Comparison of newborn care seeking behaviours (NCS) baseline (B1), survey 1 (S1) and survey 2 (S2) | Randomized at union level | NCS outside home from B1 to S2 for I1vs. C2: 92.9%vs. 93.7% to 93.2% vs. 95.3% (N/S)  NCS from qualified for I1 vs. C1 for B1: 31.2% vs. 29.6%, to S2: 60.4% vs. 33.9% (p<0.0001)  NCS from hospital I1 vs. C1 for B1: 17.9%vs. 17.6%, to S2: 46.4% vs. 23.0% (p<0.0001)  NCS from unqualified I1 vs. C1 for B1: 66.7% vs. 67.9%, to S2: 36.7% vs. 65.0% (p<0.0001) | | ++ |
| **Interventions to Promote Newborn Care** | | | | | | | | | | |
| Darmstadt 2006 | Rural Uttar Pradesh, India | Before and After nested in cRCT | Pregnant women, their families and influential community members | Pregnant women residing in study area who agreed to stay in area for at least 1 week post delivery | I1:Antenatal and postnatal home visits for BCC targeting ENC practices (BF, birth preparedness, hygiene and cord care, thermal care (STSC skin-to-skin care).  I2: I1 plus use of Thermospot - device to assess body temperature | C1: received usual health services | Randomized | Acceptability of STSC - I1 and I2 vs. C1: 74.5% for NBW, 76% for LBW vs. <10% for both NBW and LBW | | ++ |
| Quasem 2003 | Sylhet, Banglad-esh | Case Series | Expectant women (7th month gestation or greater), newly (within 7 days) postpartum and their postpartum support people | Women approx. 32 days postpartum | Pilot programme  for CKMC, using peer communication and demonstrations to promote KMC, proper cleaning of newborns, EBF on demand, and health seeking behaviours. Visual aids via flashcards and pictorial pamphlets were used. | Survey and interview of all women enrolled in CKMC intervention, 1 month postpartum. | N/A | Women’s experiences with CKMC: 77% initiated STSC.  STSC for LBW vs. not LBW: 85% vs. 73%  STSC for female vs. male: 83% vs. 74%  STSC use, taught KMC before vs. after delivery: 78% vs. 75%  STSC most of the time in first 2 days, first week, first month: 66%, 45%, 26%, respectively.  Immerse cleaning - 100%  Immerse cleaning 1st day, STSC users vs. Non-STSC: 59% vs. 75%  Upright sleeping of baby: 14%  EBF 1 month: 37%  EBF 1 month STSC users vs. Non-STSC: 41% vs. 25% | | + |
| Kumar 2008 | Rural Uttar Pradesh, India | cRCT | Pregnant women, their families and influential community members | Newborns, and usual residents of HH, in study area for 15 plus successive days during 6 mths pre delivery, women who delivered during study period | I1:Antenatal and postnatal home visits for BCC targeting ENC practices (BF, birth preparedness, hygiene and cord care, thermal care (STSC skin-to-skin care), at 2 antenatal and 2 postnatal HH visits  I2: I1 plus use of Thermospot - device to assess body temperature | C1: received usual health services | Randomized using baseline covariates for stratification (standard of living and religion) | ANC - no significant difference  Birth Preparedness - all but one outcome significant (previous identification of birth attendant for I1vsC1 RR=1.54 p=0.06)  Place of delivery - NS  Delivery attendant -NS  Immediate care - I1 and I2 vs. C1 significant difference for all measures  Thermal care - significant difference all measures  Umbilical cord care - NS for tying and cutting of cord. Significant for I1 and I2 vs. C1 for other measures  BF - I1 and I2 vs. C1 significant all measures  Care seeking - NS, except for use of unqualified lower in I1 and I2 vs. C1  Mortality reduction 54% (p=0.0001) for I1vs. C1, and 52% (p=0.001) I2vs. C1 | | +++ |
| Sloan  2008 | Dhaka and Sylhet, Banglad-esh | cRCT | Expectant and postpartum women aged 12-50 and their families | Mothers and their infants in both intervention and control | Behavioural intervention to promote CKMC, proper newborn care, and care seeking | No CKMC teaching | Stratified and randomized at village level | I1: 77.4% ever practiced CKMC, 61% w/in 12 hours birth, 23.8% STS >7h/day  I1: STS in home delivery 85.9% vs. elsewhere 59.9% (p<0.001)  CHW visit last month pregnancy, STS 87.2%  I1 breastfed 3.4h sooner than C1  Immersion bathing, I1vs.C1: 29.3%vs. 72.3%  Diarrhea I1vs.C1: 43.6% vs.39.3% (p=0.006)  No growth difference or mortality difference | | +/ |

1. #

   [↑](#footnote-ref-1)
2. From EPHPP Assessment.

   + = weak, +/ = moderate-weak, ++ = moderate, ++/ = moderate-strong, +++ = strong [↑](#footnote-ref-2)
