# Supplementary material for: Effectiveness of community health workers delivering preventive interventions for maternal and child health in low- and middle-income countries: a systematic review
Source: BMC Public Health. 2013 Sep 13;13:847. doi: 10.1186/1471-2458-13-847 (PMC3848754; doi:10.1186/1471-2458-13-847)
Supplement: Additional file 5 — Search log. Dates searched and returned results for the databases used in this review. [file 1471-2458-13-847-S5.docx]

| Additional file 5 – Excluded full text reviewed characteristics | | | | | | | | |
| --- | --- | --- | --- | --- | --- | --- | --- | --- |
| **Author and Year** | **Setting** | **Study Design** | **Population** | **Intervention** | **Control/**  **Comparison** | **CHW** | **Outcomes/**  **Outputs** | **Reason for**  **Exclusion** |
| **Alderman 2008** | Fatick, Kaolack and Kolda, Senegal | Cohort | Mothers and caregivers of young children, and pregnant women | Growth monitoring services and health promotion for children | Difference in difference comparison between the three cohorts (intervention sites) | Community health and nutrition workers | Health care and health seeking behaviours, underweight children | Intervention – multifaceted and can’t distinguish CHW household roles |
| **Arifeen 2009** | Rural Bangladesh  Beginning | Cluster randomized trial | Children under 5 | IMCI – 3 components health worker training, health system improvements, and family and community activities | No intervention in control village | Village practitioners,  Village nutritional workers, Imams | Mortality, exclusive breastfeeding, stunting | Intervention - multifaceted, IMCI  CHW – role can’t be distinguished |
| **Ashwell 2009** | Papua New Guinea 1998-2004 | Multi-methods outcome evaluation | Women and children | AusAID funded Women and Children’s Health Project for education, community development and health promotion interventions aimed to increase community support for health of women and children | Survey | Village Health Volunteers (VHV) | Healthy behaviours, use of services and health knowledge | Population – unclear  Intervention – multifaceted and prevention can not be distinguished |
| **Balaluka 2012** | DRC – Katana health district | Case Comparison | Children under 6 months | Education on breastfeeding among pregnant women and community leaders. | Compared to district with no programme | Community Volunteers | Average duration of exclusive BF. Proportion receiving EBF.  Mean weight of child | Intervention – community mobilization component |
| **Bang 1999** | India, Gadchiroli, India | Control trial | Mothers and their neonates | Package of home-based neonatal care: birth asphyxia, premature birth or low birth weight, hypothermia, breastfeeding problems, sepsis, health education. | Control area no package of care | Village Health Workers | Neonatal and infant mortality.  Neonatal sepsis | Intervention multifaceted and components can not be distinguished |
| **Bang 2005a** | Gadchiroli, India | Control trial | Mothers and their neonates | Package of home-based neonatal care: birth asphyxia, premature birth or low birth weight, hypothermia, breastfeeding problems, sepsis, health education. | Control area no package of care | Village Health Workers | SBR, NMR, PMR, PNMR, IMR | Intervention – multifaceted and components can not be distinguished |
| **Bang 2005(b)** | Gadchiroli, India | Before and after | Mothers and their neonates | Package of home-based neonatal care: birth asphyxia, premature birth or low birth weight, hypothermia, breastfeeding problems, sepsis, health education. | Baseline vs. post intervention | Village health Workers | Neonatal morbidities by cause | Intervention – multifaceted and components can not be distinguished |
| **Baqui 2008** | Bangladesh, Sylhet District | Cluster RCT | Married women 15-49 years | Home-care arm –2 antenatal visits, 3 postnatal to promote birth and newborn preparedness, assess newborns, make referrals  Community-care arm – group sessions on birth and preparedness by qualified professionals | No intervention – | Female CHWs | Neonatal mortality. Intention to treat | Intervention - multifaceted and components can’t be distinguished |
| **Baqui 2008b** | India,  Rural Uttar Pradesh | Quasi-experimental design | Women who had given birth in the 2 preceding years | Integrated Nutrition and Health Programme | No intervention district | Anganwadi workers, auxiliary nurse-midwives and change agents | Frequency of home visits  Neonatal mortality | CHWs can’t be distinguished |
| **Baqui 2009** | Bangladesh, Sylhet District | Observational Cohort | Married women 15-49 years | Home-care arm –2 antenatal visits, 3 postnatal to promote birth and newborn preparedness, assess newborns, make referrals  Community-care arm – group sessions on birth and preparedness by qualified professionals | No intervention | Female CHWs | Assess effect of timing of first postnatal home visit on  Neonatal Mortality | Intervention – multifaceted and prevention can not be distinguished |
| **Bari 2006** | Bangladesh, Tangail District | Before and After of a Cluster RCT | Married women of reproductive age registered and monitored for pregnancy | Improving maternal and newborn conditions by a)behavioural change communication b) identification and referral of the sick c) strengthening of neonatal care in health facilities, by CHWs delivering care at the home | Comparison arm with no intervention | CHWs | Referral of sick, compliance after referral by CHW, care seeking from qualified providers | Study – CHWs ability for referral, referral compliance |
| **Bashour 2008** | Syria,  Damascus | RCT | Women who recently gave birth recruited from hospital | Home visits aimed to examine, follow up, educate, support and counsel by registered midwives | Group A- 4 visits  Group B – 1 visit  Group C – no visits | Registered Midwives | Maternal and neonatal morbidity and mortality | Non-CHW |
| **Bhandari 2004** | India, Haryana state | Cluster RCT | Infants living locally, and born within 9 months of training | Promoting messages, assessment of feeding practices, identification of difficulties, information provided on the benefits of exclusive BF. | No community based intervention | Health and Nutrition workers, Anganwadi workers, auxiliary nurse midwives and other health providers | Exclusive BF rates  Diarrhea morbidity and anthropometry at 3 and 6 months | CHWs - roles can’t be distinguished |
| **Bhandari 2012** | India, Haryana State | Cluster RCT | Infants born within 9 months of training | CHWs training to conduct postnatal home visits and women’s groups meetings; physicians, nurses and CHWs trained to treat or refer sick infants and children, health services strengthened | No community intervention | CHWs  Midwives, Anganwadi workers, nurses | Neonatal and infant mortality. Newborn care practices | CHWs – roles can’t be distinguished |
| **Bhutta**  **2008** | Pakistan | Cluster RCT | Participants in the governments regular LHW programme | LHWs received additional training, conducted community education group sessions, and to work with Dais  Dais received 3 day (voluntary) training | 4 village clusters – no additional training, community education or linking with Dias | Lady Health Workers  Dais | Stillborn rate, neonatal mortality, skilled attendance at birth, child care education | Intervention – includes community mobilization |
| **Bisimwa 2009** | DRC | Cross sectional | Children under 5 | CV monitored child’s grown via weight-for-age | N/A | Community Volunteers | Effectiveness of CVs in monitoring growth of U5 | Intervention – includes community sessions |
| **Brugha 1996** | Ghana, Eastern region | Controlled trial | Children 12-18mths in intervention clusters | Survey of children’s immunization status, followed by home visits for those who failed to complete immunization targets | Control group received no visits | 0-Level graduates and nurses | Immunization coverage | CHWs – do not meet definition |
| **Chanda 2011** | Zambia, Chongwe district | Prospective evaluation | All people living in area | Delivery of RDT tests and ACT by CHWs and referral or treatment appropriately | N/A | Community Health Workers | Efficiency of CHWs as delivery points for ACT and RDY in the home management of malaria | Intervention – not MCH specific |
| **Chudasame 2009** | India, South Gujarat | Cross sectional evaluation | Children under 5 | Booth activity and house activity of IPPI | N/A | ANM, FHW, Anganwadi workers, Social health activists | People living in catchment areas awareness of IPPI, and source of information | CHWs- roles can’t be distinguished |
| **Cisse**  **2009** | Senegal, Ndoffane district | cRCT | Children 3-59 months | Children received one of 3 drug combinations for malaria, once a month during transmission season, delivered by CHWs | Comparison of the three different drug combinations | Community Health Workers | Malaria incidence and adverse events | Study – on effect of different malarial drugs in children |
| **Coetzee 1993** | South Africa,  Alexandra township | Cross sectional Impact evaluation | Children 12-23 months | Child health outreach programme for vaccination through outreach points integrated with well baby clinics | N/A | Nurses | Guardian’s knowledge of vaccination, attendance at postnatal clinic, use of outreach services | CHWs – nurses are used |
| **Connor 2011** | Malawi,  South | Retrospective analysis | Moderately malnourished children enrolled in selective feeding programme, between 6-60 months | Home based therapy (HBT) using ready to use foods (RUTF), with MUAC progress being monitored by community-based health aids | Body weight changes experienced during treatment and changes in arm circumferences over same time  Health-surveillance assistants | Village health aides  Community-based health aids | Ability of field measurements of MUAC to track body changes during intervention in a home based setting | Intervention – not prevention |
| **Darmstadt 2009** | Bangladesh, Mirzapur | Cross sectional within an RCT | Neonates | CHWs trained to conduct household surveillance and identify and refer sick newborns, as part of a larger community based trial | Looked at all live-born neonates enrolled and did CHW assessment | Community Health Workers | Referral rates, parents adherence to referral, CHW visits to neonates | Intervention – not prevention |
| **Darmstadt 2009** | Bangladesh | Cross-sectional | Neonates | CHWs evaluated breastfeeding and signs and symptoms of illness in neonates. Physicians independently evaluated all neonates to validates CHW identification | CHWs compared to physicians | Community health workers | Validity of CHW assessment of neonates illness and breastfeeding | Intervention – not prevention |
| **de Haas 1994** | Indonesia, North Sulawesi | Descriptive and questionnaire | Pregnant women and neonates | CHWs and TBAs assessed for knowledge of tetanus intervention | N/A | Community health workers  TBAs | Knowledge of tetanus and prevention | Intervention – CHWs ability/knowledge |
| **Dickerson 2010** | Tibet | Descriptive and cross sectional | Pregnant women at rick of having unattended pregnancy and their babies | MCH education, simple life saving skills, and distribution of clean birthing kits, essential newborn clothes and micronutrient supplements. | NA | Lay persons | Number of mothers who received outreach, education, training, and supplies | UN – not clearly defined as CHWs, and hard to distinguish roles |
| **Dongre 2009** | India, Wardha | Before and after | Mothers of newborns, pregnant women | Community based participatory intervention for newborn health care seeking | Baseline survey compared to 3 years after initiation of intervention | CLICS doot (female community health worker), trained social workers | Mothers knowledge on newborn safety, proper treatment, and where they got their information | CHWs – can’t distinguish role |
| **Ebuehi**  **2010** | Nigeria,  Osun State | Cross-sectional | Mothers or caregivers of children 0-59mths, and their index children | IMCI (improved case management, improved health systems support, improved family and community practices known as C-IMCI | C-IMCI-compliant Local government area (LGA) vs. LGA where C-IMCI was not yet implemented | Community resource persons (CORPs) | Disease prevention including: Hand-washing, malaria prevention and HIV/AIDS prevention | Intervention – IMCI  CHW – roles not defined |
| **Emond 2002** | Brazil, Natal | Before and after surveys | Community | ProNatal project – integrated community health | N/A | Health agents – recruited from local community | Population health statistics | Intervention – Not specific MCH, prevention aspect can not be distinguished |
| **Feldens 2007** | Brazil,  Sao Leopoldo | RCT | Mothers who gave birth within the public health systems | Home advice 10 days after birth, then once a month until 6 months, again at 8,10 and 12 based on “Ten Steps for Health Feeding” | Control group received routine assistance by pedestrians | Fieldworkers - trained | Early childhood caries (ECC) at 12 mths | CHWs – “fieldworkers” not defined but don’t appear to fit definition |
| **Flynn-O’Brien 2011** | Kenya | Before and After | Caretakers of children under 5 | Programme to increase appropriate care and treatment for malnutrition and diarrhea prevention and treatment | 2007 baseline compared to 2009 randomly selected districts with cross sectional survey | Community Health Worker - CHW | Prevalence of malnutrition, child morbidity, KAP of caregivers, EBF | Report – Conference abstract, unable to find related article |
| **Ghimire 2010** | Nepal | Review | Children under 5 | Community based interventions for under 5s for diarrhea and ARIs | Results from 20 years | Community health volunteers (CHVs) | Reporting of diarrhea and ARIs, proportion with dehydration and pneumonia, case fatality rates | Study – review, can’t distinguish CHWs role |
| **Gies**  **2008** | Birkina Faso, Boromo | Control trial | Pregnant women | A)Community promotion in addition to IPTp-SP (4 health centers).  B) 4 centers assigned IPTp-SP | 4 centres assigned weekly chloroquine | Female community leaders - | ANC attendance and SP uptake | Intervention – community based health education not described |
| **Gupta**  **1991** | India | Cross-sectional | 619 children, 6 wks – 2 year) | Assessment of children by Anganwadi workers using WSST (Woodside system so screening) for development issues – physical, mental or sensory ability. For early detecting of impairment | All results compared with that of a trained professional | Anganwadi Workers, pre-trained, non professional | Reliability of Anganwadi workers to test for development issues | Intervention – CHW ability/knowledge |
| **Gupta**  **2011** | India, Tamil Nadu | Cross sectional comparison | Randomly selected households | Behaviour change aimed at preventing diarrheal illness through home visits, community events and health education via CHWs | Control population not enrolled in program | Community health Worker | KAP of program related to diarrheal illness | Intervention – not MCH specific |
| **Harvey 2008** | Zambia, Lusaka Province | Control trial | CHWs | Ability to prepare RDT to test of malaria using three different methods | 3 groups of CHWs: 1) prepared RDT using manufactures instructions 2) CHWs used only job aid 3) RDT using job aid after and three hours of training | Community Health Workers | CHWs ability to use Rapid Diagnostic Tests (RDTs) for malaria safely and effectively | Intervention – CHWs ability/knowledge, plus not specific to MCH |
| **Hawkes 2009** | DRC | Cross sectional | CHWs on febrile children,  0-14 years | CHWs trained to safely and accurately perform and interpret RDTs, then perform on febrile children | CHWs surveyed with questionnaire for knowledge and skills | Community health workers, trained for 1 day | If CHWs can safely and effectiveness use RDTs and cost -effectiveness | Intervention – Not child under 5 and CHW ability |
| **Hoare 1994** | The Gambia | Descriptive | Women with babies 4-9 mths | Weaning and adapted food for better nutrition demonstrations | Dietary assessment forms | Village women as teachers | Adaptation of local foods to improve nutritional content | Intervention – community demonstrations |
| **Hodgins 2010** | Nepal | Before and after impact study via three stage cluster sampling | Pregnant women and family members | Community based antenatal counseling (the Birth Preparedness Packages) and dispensing (Iron/folate) and an early postnatal home visit (iron/VitA) | Pre and post intervention surveys | Female Community health volunteers | Household practices and service utilization  - (danger signs, nutrition, essential newborn care) | CHWs – can’t distinguish role |
| **Jennings 2011** | Benin | Cross sectional, | Lay nurse aids on MCH | Nurse-midwives and lay nurse aids trained to use job aids to improve counseling in MCH | Lay nurse aids quality vs. nurse-midwives | Clinic based lay nurse aids | Quality of counseling for MCH | Intervention – not home based  CHWs – do not fit definition |
| **Jetten 2010** | India, Indore | Experimental before and after pilot study | Families with it least 1 child under 4 | Educational program in combinations with the distribution of a barrier or playpen to decrease domestic burns in young children | Before and after | Unknown – seems as if the researchers did the intervention | Number of burns reported, frequency of dangerous house situations | CHWs – not used |
| **Khanal 2011** | Nepal, Morang district |  | Neonates and young infants | CHWs to classify sick young infants with possible severe bacterial infection. FCHV home visits after delivery – found infants with PSBI, treated and referred | Assessment by FCBV compared to assessment by more qualified | Female community health volunteers and paid facility-based community health workers | If CHWs can effectively and correctly follow a set of guidelines to identify possibly severe bacterial | Intervention – testing CHW ability |
| **Khresheh 2011** | Jordan, southern | RCT | Women, first time pregnant, | Education program supporting breastfeeding, offered a one-to-one postnatal education session, and follow-up phone calls at 2, 4 mths. | Control group received routine postnatal care | Researcher | BF knowledge | CHWs – not used  Intervention – not home based |
| **Kidane 2000** | Ethiopia, Tigray | RCT | Mothers of children under 5 | Teaching mothers to promptly provide antimalarials to sick children | Control, mother coordinators not taught to train moms | Mother coordinators | Under 5 mortality from malaria | Intervention – teaching to treat malaria |
| **Le Roux 2010** | South Africa | RCT | Mother-child dyads, with underweight children under 5 | Home visiting program to improve childhood nourishment where | Control group with no Mentor Mothers intervention | Paraprofessional Mentor Mothers | Timing of rehabilitation from malnourishment | Intervention – rehabilitation |
| **Lee 2008** | Burma, Eastern conflict areas | Descriptive/  Report | VHWs | Trained in comprehensive set of interventions for malaria control | N/A | IDPs trained as Village health workers | Capability of IDPs as VHWs for malaria interventions | Intervention – not MCH specific |
| **Leite 2005** | Brazil, Fortaleza | Randomized Clinical Trial | New mothers and newborns | Breastfeeding counseling during home visits on days 5,15,30,60,90 and 120 days by lay counselors | Control group – no home visits | Lay counselors from the community | Exclusive BF, delayed utilization of formula | CHWs – do not fit with definition |
| **Malekafzali 2000** | Iran, Bakhtiari province | Before and After | Children under-5 | Nutrition, deworming and sanitation education and support for growth monitoring programme | One year post intervention initiation | Behvarz and volunteer women | Mother’s KAP, breastfeeding practices and child malnutrition, | Intervention – not delivered exclusively through household |
| **Mahmood 2010** | Pakistan, district Lahore | Cross sectional descriptive | Lady Health Workers | Survey for LHWs in reporting on family planning and primary health care. | N/A | Lady Health workers whom had completed formal training and been working for it least 1 year | Quality and accuracy of data recording and reporting tools. | Intervention – Ability/quality of CHWs |
| **Manandhar 2004** | Nepal, Makwanput district | Cluster RCT | All women in study area | Nine action learning women’s group meetings every month | No women’s groups | Female facilitator | Birth outcomes, uptake of services, home care practices, infant morbidity and health care seeking. | Intervention – community groups |
| **Mathur 1995** | India, Gorakhpur | Cross sectional and longitudinal follow up | Children below 6 | Anganwadi workers detect disability in children under 6 | Confirmed by doctor | Anganwadi workers in an Integrated Child Development Service (ICDS) | Ability of AWW to identify and manage disability | Population – Children under 6  Intervention - CHW ability |
| **McPherson 2006** | Nepal, Siraha | Before and after | Mothers and infants under 1 | CHWs promoted a birth-preparedness package (BPP) through an inter-personal counseling with individuals and groups. | Baseline compared to end line | Female community health volunteers, trained TBAS | Effectiveness of BPP to positively influence planning for births, HH behaviours, and use of services | CHWS – role can’t be distinguished |
| **Mens**  **2011** | Nigeria, Edo State | Before and after survey | Women of child bearing age | Peer education to raise knowledge of malaria during pregnancy and its effects on mother and fetus on women in child bearing age, through workshops, rallies, and door-to-door campaign | Before and After intervention using questionnaire | Peer educators (women) - selected from the clusters participated in study. Trained for 3 days | Women in reproductive age knowledge malaria during pregnancy | Population – all women within reproductive age  Intervention – community mobilization included |
| **Midhet 2010** | Pakistan, Balochistan | Randomized trial | All women in area | Women provided information on safe motherhood through pictorial booklets and audiocassettes, TBAs trained in safe delivery, emergency transport systems set up. | Control cluster had no intervention | Female volunteers from each village to train as IEEC facilitators | Prenatal care, hospital deliveries, safe motherhood indicators, mortality | CHWs – role can’t be distinguished |
| **Mosha**  **2005** | Tanzania, Mwanza | Stepp-wedge randomized community trial | Pregnant women age 18-45 | Clean delivery kits distributed to women on ANC visit and education based on the “six cleans” by WHO. | No clean kit intervention but regular antenatal services | Maternal and Child Health Aide | Cord infection and puerperal sepsis. Acceptability of kits | Intervention – initial phase delivered at ANC visit |
| **Msyamboza 2009** | Malawi, southern | Control trial | Pregnant women | Education and counseling on malaria control in pregnancy and the importance of attending antenatal clinics. Distribution sulfadoxine-pyrimethamine (SP). | Control received CHW education but no SP | Female village-based Community health workers | Coverage of IPPT. | Study – evaluating effect of SP, not CHW |
| **Mushi**  **2010** | Tanzania,  Mtwara district | Before and after | Pregnant women and their families | Promotion of early and complete antenatal care visits and delivery with skilled attendant | 2 years post intervention initiation | Safe motherhood promoters (SMPs) 4 TBAs were included | Delivery with skilled attendant. ANC bookings. Continuation of SMP intervention | CHWs – TBAs also included and role can not be distinguished |
| **Nair**  **2009** | India, Kerala | Before and after | Mothers of children 0-6 years | Oral health education materials delivered in classes | Before and after survey on knowledge | Junior Public Health Nurses (JPHNs) and Anganwadi workers (AWWs) | Effectiveness of community oral health awareness programme | Intervention – not HH based  CHWs – also included junior nurses |
| **Olusanya 2008** | Nigeria, Lagos | Cross sectional | Infant 3 months or under attending BCG clinics | Two stage screening protocol for infant hearing using TEOAE and AABR by health workers at children attending BCG clinics | N/A | Staff members – 2 full time and 2 part time. | Screening  sensitivity, specificity, PPV and NPV | Intervention – at immunization clinic  CHWs – include trained hospital staff |
| **Omer**  **2008** | Pakistan, Sindh Province | Pilot Randomized Control Cluster Trial | Pregnant women in communities | LHW used new tools based on culturally appropriate interaction around relevant evidence for child health promotion | Control group – LHWs used standard procedure | Lady Health Workers | Effectiveness of community-based evidence for health promotion of MCH by LHWs | Intervention – target not prevention |
| **Pence**  **2007** | Ghana, Navrongo | Four-arm community-randomized controlled experiment | Children under 5 | Promotion and education of basic childhood health issues using 3 arms 1)Community (CHWs) 2)MOH (nurses) 3) Community and MOH | Comparison receives services according to standard MOH guidelines. | Health volunteers – appointed by village health committees, | Health and mortality impact of three alternative organizational strategies for children under 5 | Intervention – CHW arm involves community groups |
| **Perez**  **2009** | Mali | Cross sectional using multi-stage cluster sampling | Households in area, interviews with caregivers of children under 5 | Promotion of household and community health practices through CHWs | N/A | Community Health Workers (CHWs) | Assess performance of CHWs in promotion of basic child heath services | Intervention – not specific MCH |
| **Powell**  **2004** | Jamaica | Cluster RCT | Underweight children 9-30 mths | Weekly visits demonstrating playing and healthy child stimulation | No visits encouraging healthy parenting | Community Health Aids | Effect on children’s development, mother-child relationship and mother’s knowledge | Intervention – rehabilitation |
| **Prasanta 2010** | India, Orissa and Jharkhand | Cluster RCT | Women 15-49 that had given birth during study | Women’s groups to support participatory action-learning, and development and implementation of strategies to address MCH problems. | Control had no women’s groups | Facilitator | Neonatal mortality, maternal depression | Intervention – women’s groups |
| **Rotheram-Borus**  **2011** | South Africa, Cape Town | RCT baseline sample | Pregnant mothers at risk for HIV/alcohol and/or nutrition problems | Four antenatal and 4 postnatal home visits that address HIV, alcohol, nutrition, depression, health care regimes, caretaking and bonding, and securing government grants. | Control of standard care of neighbor clinic based services | “Mentor Mother” CHWs recruited from township neighborhoods trained for intervention | Ability of CHWs to reach mothers, household and mother characteristics | Study – baseline survey |
| **Sievers 2008** | Rwanda | Retrospective Before and After | Pediatric admissions for malaria | Two-pronged approach to combating malaria via mass distribution of insecticidal treated nets and distribution of antimalarial medications by CHWs | I.D. of laboratory confirmed malaria, suspected malaria, all-cause admissions. | Community Health Workers | Impact of interventions on pediatric hospitalizations and on laboratory markers of malaria. | Intervention – treatment can’t be distinguished  CHWs – role can’t be distinguished |
| **Singh**  **2009** | India | Before and after, cross-sectional | Women within reproductive age | Community based intervention to reach women direction with information and support for accessing safe abortions. | Before and after monthly client load in two public and two private facilities | CHWs, auxiliary nurse-midwives, TBAs and outreach workers | Impact of intervention on client load at safe service facilities | Intervention – community mobilization  CHWs – role can’t be distinguished |
| **Stromberg 2011** | Kenya | Before and after survey | Mothers of children under 5 | CHWs provide educational sessions and Insecticide-treated nets (ITNs) to mothers | Baseline survey 9 mths after initiation of intervention | Community health workers (CHWs) | Families knowledge of malaria and use of ITNs for children under 5 | Intervention – group education sessions |
| **Stwart**  **2008** | South Africa | Cluster RCT | Households with children under 10 | Home visitation program to improve home safely and prevent injuries among children | Control – no safety intervention | Paraprofessional | Presence of home hazards for burns, poisoning and falls. | Population – children under 10 |
| **Taylor**  **2001** | KwaZulu-Netal | Before and after | Households | CHWs delivering nutrition information at household level and community meetings | Baseline survey and survey 6 months after initiation | Community Health Worker | Household perceptions of intervention | Population – not specific MCH |
