# Supplementary material for: Effectiveness of community health workers delivering preventive interventions for maternal and child health in low- and middle-income countries: a systematic review
Source: BMC Public Health. 2013 Sep 13;13:847. doi: 10.1186/1471-2458-13-847 (PMC3848754; doi:10.1186/1471-2458-13-847)
Supplement: Additional file 6 — Excluded full text reviewed characteristics. Additional information on the studies that had their full texts reviewed, including the reason they were excluded from this review. [file 1471-2458-13-847-S6.docx]

### Additional file 4 – Search log

| **Database** | **Date Searched** | **Total Results** |
| --- | --- | --- |
| Web of Science | June 8^th^, 2012 | 1,815 |
| Embase | June 10^th^, 2012 | 1,972 |
| PubMed | June 10^th^, 2012 | 375 |
| Scopus | June 10^th^, 2012 | 1,675 |
| CINAHL | June 11^th^, 2012 | 2,690 |
| OVID Nursing | June 11^th^, 2012 | 1,547 |
| POPLINE | June 11^th^, 2012 | 147 |
