# Supplementary material for: Effectiveness of community health workers delivering preventive interventions for maternal and child health in low- and middle-income countries: a systematic review
Source: BMC Public Health. 2013 Sep 13;13:847. doi: 10.1186/1471-2458-13-847 (PMC3848754; doi:10.1186/1471-2458-13-847)
Supplement: Additional file 7 — CHW characteristics. Characteristics of the community health workers for each of the included, including training, supervision, and other factors that should be considered when analysing CHW programmes. [file 1471-2458-13-847-S7.docx]

| Additional file 6 – CHW characteristics | | | | | | | |
| --- | --- | --- | --- | --- | --- | --- | --- |
| Article | Description | Requirement | Recruitment | Training | | Supervision | Ratio^[[1]](#footnote-1)^ |
|  |  |  |  | Initial | Additional |  |  |
| Ahorlu2009 | Community Assistants | Un | Community members | Un | Un | Un | Un |
| Okeibunor 2011 | Community -directed distributors  Volunteer | No specific requirement, preference given to women from each local kindred with childbearing experience | Selection by each kindred within communities. Over 700 from more than 450 kindred groups | Training to deliver ITNs and SP, and basic counseling services by staff of nearest health facility | Un or N/A | Frequency unknown.  Provided by staff at nearest health facility | 1:120 (women of child-bearing age) |
| Darmstadt 2006 | Community Health Workers, *Saksham sayahak*  Volunteer | Un | Majority were beneficiaries of intervention whom subsequently volunteered | Un | Un | Un | 1:3500 population |
| Kumar2008 | Community Health Workers, *Saksham sayahak* | 12 years or more education, proficient communication and reasoning skills, references from community stakeholders | From local community, referred by key stakeholders | Classroom and apprentice-based combination | Mentored by programme supervisor for additional week before initiation | Performance feedback from community members, monitoring by supervisors for meeting targets | Un |
| Sheth2004 | Anganwadi workers | Provide description | get this somewhere else | MOH regular training plus food safety education in 2 sessions using resource material and action based learning | Un | Gov vs. this study?? | 1:25 enrolled |
| Owais2011 | Community health workers | Local women | Un | Un | Un | Un | Un |
| Haider2000 | Peer Counselors  Part-time, paid US$22.50/mth | Women with at least 4 years schooling and personal breastfeeding experience, residing in intervention zones | One peer counselor per intervention zone | Components of WHO/UNICEF BF counseling course and King’s book as guides. 40 hours, over 10 days. Demonstrations, role play and contact with mothers. 2 week pilot counseling skills practiced. | Un | Monitored at least three times over study period by supervisors | 1: 12-25 enrolled |
| Cooper2009 | Lay community workers | Women, no formal qualifications (no education required, but 2 of 4 had completed schooling) | Selected with help from local community council. | Training over four months on basic parenting, counseling and the specific intervention. | Weekly supervision also offered session by session information | Community clinical psychologist for group supervision weekly | Approx. 1:120 enrolled |
| Rahman 2008 | Lady Health Workers | Completed secondary school | Already trained Pakistani LHW | Regular LHW training, plus extra 2 days of training for intervention | 1 refresher training day after 4 months of intervention | Half-day group supervision occurring monthly by experienced member of research team. | 1:100 HH normal, plus 1:12 |
| Bari2006 | Community Health Workers | Minimum 10th grade education, residing in population they serve | Un | One month initial training for basic package of maternal and newborn care |  | Daily activities supervised by Field Supervisors. Review of key performance and process indicators each fortnight. | 1:4000 population |
| Brenner 2011 | Volunteer community health workers, paid $1.50/mth for transport, several basic non-financial incentives | Selected by community | CHW selection meetings held in villages, where after description of role and village expressed expectations, 2 individuals nominated | 5 day course stressing C-IMCI guidelines held in each village. Training via “train the trainers” where local health center staff with a variety of backgrounds trained to train CHWs. | 2 hour refresher each month during monthly meeting | Monthly meeting with trainers/supervisors throughout study | 1:45 children under-5  (1:25HH) |
| Tylleskar 2011 | Peer Counselors | Living in or near intervention clusters | Un | One week training by national teams, using WHO courses: Breastfeeding counseling: a training course, and HIV and infant feeding counseling: a training course | Study supervisor visits fortnightly for mentoring | Study supervisor visits fortnightly (mentoring) and supervision | Un |
| Quasem 2003 | Community-based nutrition promoters (CNPs), paid approx. $0.50/month | Female community residents. Have seventh grade or higher education level. | CNPs already working with Bangladesh Integrated Nutrition Programme (BINP) were selected for additional training. | One week training: 2 days background, 1 day supervised hand-on practice, 1 day role-playing, 2 days supervised initiation | Periodic meetings over 8 weeks for sharing of experiences | Two supervisors. Last 2 days of training were supervised visits, attended regular meetings during study and supervisors conducted unannounced visits to observe CKMC teaching by CNPs | 1:1-2 enrolled |
| Sloan2008 | Community nutrition workers, paid $7.50/mth | (Assumed) met NNP requirements of seventh grade or higher education | Not explicitly stated but assumed already members of National Nutrition Programme (NNP) | Two month training period of CHWs and their supervisors | Monthly refresher training sessions | Un | 1:210 enrolled |
| Agrasada 2005 | Barangay (village) health volunteers, received transport costs for home visits and training | Already Barangay health volunteer, positive personal experience with breastfeed.  Between 22-50, similar education to mothers. | Postnatal care seminar for Barangay workers, where women were then approached | Forty hours of interactive and practical training by certified lactation counselor | Un | Lactation counselor monitored throughout study | 1:5 enrolled |
| Mannan 2008 | Community health workers | Un | From community | Twenty-one days of training (classroom, hospital and community based) including approx. 2.5 days practical. | Monthly refresher training sessions | Field supervisor (1 for 8 CHWs), observed at least 2 full days/month, assessed using structured checklist, and provided written feedback | 1:4000 population |
| Morrow 1999 | Peer counselors | Un | Recruited from same community as intervention | By La La Leche League (International NGO for breastfeeding promotion) | Un | Un | Un |

1. Approximate number of individuals (unless unit stated differently) each CHW is responsible for. Represented as ratio of CHW to Individual [↑](#footnote-ref-1)
